# Interdependent recruitment of CYC8/TUP1 and the transcriptional activator XYR1 at target promoters is required for induced cellulase gene expression in *Trichoderma reesei*

**DOI:** 10.1371/journal.pgen.1009351

**Published:** 2021-02-19

**Authors:** Lei Wang, Weixin Zhang, Yanli Cao, Fanglin Zheng, Guolei Zhao, Xinxing Lv, Xiangfeng Meng, Weifeng Liu

**Affiliations:** State Key Laboratory of Microbial Technology, Microbial Technology Institute, Shandong University, Qingdao, Shandong, People’s Republic of China; University College Dublin, IRELAND

## Abstract

Cellulase production in filamentous fungus *Trichoderma reesei* is highly responsive to various environmental cues involving multiple positive and negative regulators. XYR1 (Xylanase regulator 1) has been identified as the key transcriptional activator of cellulase gene expression in *T*. *reesei*. However, the precise mechanism by which XYR1 achieves transcriptional activation of cellulase genes is still not fully understood. Here, we identified the TrCYC8/TUP1 complex as a novel coactivator for XYR1 in *T*. *reesei*. CYC8/TUP1 is the first identified transcriptional corepressor complex mediating repression of diverse genes in *Saccharomyces cerevisiae*. Knockdown of *Trcyc8* or *Trtup1* resulted in markedly impaired cellulase gene expression in *T*. *reesei*. We found that TrCYC8/TUP1 was recruited to cellulase gene promoters upon cellulose induction and this recruitment is dependent on XYR1. We further observed that repressed *Trtup1* or *Trcyc8* expression caused a strong defect in XYR1 occupancy and loss of histone H4 at cellulase gene promoters. The defects in XYR1 binding and transcriptional activation of target genes in *Trtup1* or *Trcyc8* repressed cells could not be overcome by XYR1 overexpression. Our results reveal a novel coactivator function for TrCYC8/TUP1 at the level of activator binding, and suggest a mechanism in which interdependent recruitment of XYR1 and TrCYC8/TUP1 to cellulase gene promoters represents an important regulatory circuit in ensuring the induced cellulase gene expression. These findings thus contribute to unveiling the intricate regulatory mechanism underlying XYR1-mediated cellulase gene activation and also provide an important clue that will help further improve cellulase production by *T*. *reesei*.

## Author summary

Originally identified in budding yeast, CYC8 (also known as SSN6)/TUP1 (also known as RCOA/RCO1) is one of the best studied corepressors and has served as a model for the study of similar corepressor complexes in higher eukaryotes. Besides its well-established role in transcriptional repression, CYC8/TUP1 has been reported to be involved in transcriptional activation following release from repression in *S*. *cerevisiae*. In filamentous fungi, there have been also reports on TUP1 homolog functions in specific gene repression as well as vegetative growth and asexual spore production. However, CYC8/TUP1 function in gene activation has not been described. In this study, we reveal a coactivator function for TrCYC8/TUP1 in initiating the cellulolytic response in *T*. *reesei*. TrCYC8/TUP1 itself is specifically recruited to cellulase gene promoters in an XYR1-dependent manner. We further show that TrCYC8/TUP1 contributes to the induced cellulase gene expression by improving the binding of the essential transcriptional activator XYR1. These results thus implicate a synergistic mechanism in ensuring the efficient recruitment of XYR1 and TrCYC8/TUP1 to target gene promoters.

## Introduction

The coordination of complex cellular processes from growth and differentiation to responses to environmental changes usually depends on the stringent regulation of gene expression mainly at the level of gene transcription. Transcriptional activation generally involves binding of transcriptional activators to specific DNA sites, recruitment of RNA polymerase and its associated factors, and the initiation of transcription [1]. On the other hand, the specific repression of a gene (or a set of genes), even when the necessary activators are present, usually involves the similar interaction of transcriptional repressors with recognition sites on DNA and the following direct or indirect blockage of transcription initiation [2, 3]. In eukaryotes, both processes are often accompanied by local changes in the chromatin structure brought about by ATP-dependent nucleosome-remodeling activities and histone modifying activities, both of which can be recruited to chromatin DNA by the activator or the repressor [4–6]. Central to the eukaryotic repression system is the well established yeast CYC8/TUP1 corepressor complex [7]. Although CYC8 and TUP1 sequence homologs have not been found in higher eukaryotes, corepressor proteins with similar domain structures and functions are present in flies and vertebrates [8]. Various repressors including *S*. *cerevisiae* MIG1, a *Trichoderma reesei* CRE1 ortholog, have been shown to recruit CYC8/TUP1 to turn off subsets of genes [9–12]. Once recruited to the promoter, CYC8/TUP1 has been proposed to be able to mediate the organization of nucleosomes into repressive chromatin structures [13, 14], to shield up the activation domain of transcriptional activators to block it from recruiting transcriptional coactivators [3], or to interfere with assembly of the preinitiation complex (PIC) via its interaction with mediator subunits [15–17]. Contrary to its well-established corepressor function, CYC8/TUP1 has been implicated in gene activation in *S*. *cerevisiae* [18, 19]. Thus, instead of being evicted from target promoters upon activation, the CYC8/TUP1 complex remains bound to the promoter under activating conditions and participates in the recruitment of other coactivators including SAGA histone acetylase cofactor complex and SWI/SNF ATP-dependent chromatin remodeling complex to counteract its own repressive function [18, 19]. There is also evidence that CYC8 and TUP1 can function as conventional coactivators at *cyc1*, *suc2* and *fre2* genes in *S*. *cerevisiae* although the exact mechanism is not clear [20–22]. Notably, binding of the GCN4 transactivator to amino acid biosynthetic genes *arg1* and *arg4* in *S*. *cerevisiae* is promoted by CYC8/TUP1 under conditions of starvation for any amino acid, which in turn enhances the binding of the complex itself [23].

The filamentous fungus *T*. *reesei* is an excellent producer of (hemi) cellulases for degrading renewable lignocellulosic biomass to produce environment-friendly bio-based chemicals including biofuels [24, 25]. When exposed to cellulose, *T*. *reesei* rapidly expresses and secretes large amounts of hydrolytic enzymes to synergistically degrade the insoluble cellulosic material into cello-oligosaccharides or glucose [25]. Cellulase production in *T*. *reesei* is thus in a very energy-efficient manner that is governed by both inducer-dependent expression and glucose-mediated repression of the involved cellulase genes [26]. Other environmental cues and physiological status such as light, pH and calcium have also been shown to act upon this carbon source-regulated enzyme production [27–29]. These signals and their initiated intracellular signal transduction pathways intervene to form a robust but intricate regulatory network controlling cellulase gene expression in *T*. *reesei*. Several downstream regulatory factors have been extensively studied and proved to be responsive to these environmental cues. Among others, Xyr1 (Xylanase regulator 1) is crucial to activate the expression of most cellulases and hemicellulases [30]. In contrast, CRE1 is the main transcription factor mediating carbon catabolite repression (CCR) of cellulase gene expression [31]. Accordingly, while a release from CCR has been found to be a useful prerequisite for the industrial exploitation of *T*. *reesei* for hydrolytic enzyme production [32, 33], constitutive expression of XYR1 or expression of a C-terminal A824V mutant XYR1 from its endogenous locus results in a glucose-blind hyperproduction of cellulases, in line with its key role in controlling cellulase gene expression [34–37]. Despite these findings, the exact mechanism by which XYR1 activates cellulase gene transcription and the underlying intricate regulatory network awaits further deconvolution.

In this report, we show that *T*. *reesei* orthologs of TUP1 and CYC8 form a stable complex *in vivo*. *Trtup1* or *Trcyc8* knockdown severely impaired the induced cellulase biosynthesis. We present evidence that TrTUP1 is recruited to cellulase gene promoters on cellulose induction in an XYR1-dependent manner. On the other hand, TrCYC8/TUP1 is required to promote XYR1 occupancy at the same promoters, which may provide a synergistic mechanism to achieve efficient XYR1 binding and its activation required for cellulase gene expression.

## Results

### TrCYC8/TUP1 exists as a stable complex in *T*. *reesei*

**Fig 1:**
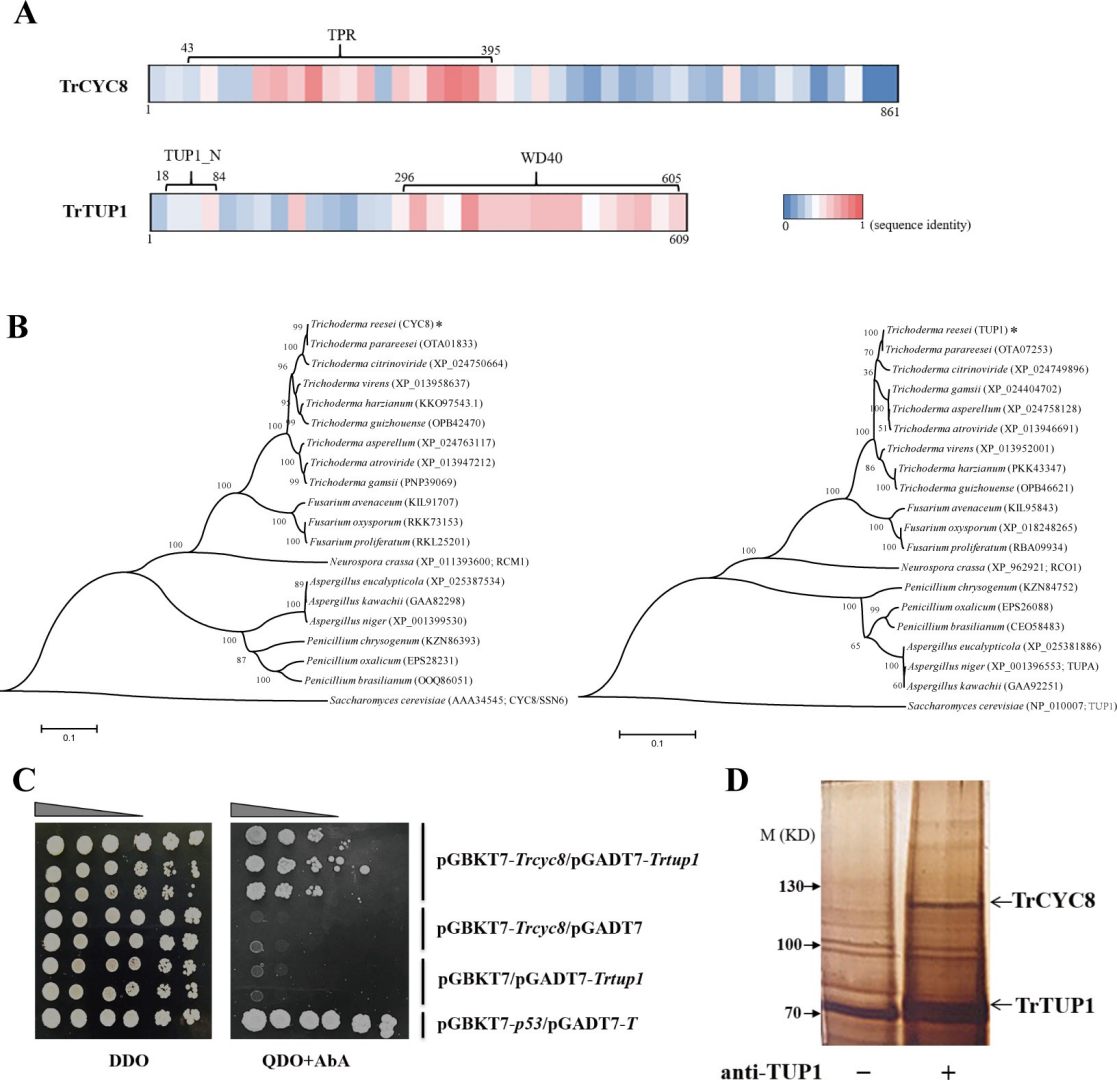
Evolutionarily conserved CYC8/TUP1 complex exists in *T*. *reesei*. **(A)** Diagram of TrCYC8/TUP1 sequence organization. The TPR domain of CYC8 and WD40 as well as TUP1_N domain of TUP1 were labeled as indicated. The respective sequence identity of TrCYC8/TUP1 relative to ScCYC8/TUP1 was illustrated as a color code in a sequence window of 20 amino acids. (B) Phylogenetic analysis of TrTUP1 and TrCYC8 orthologs. Phylogenetic trees were generated by the Neighbor-joining method using retrieved protein sequences using MEGA6. TrCYC8 and TrTUP1 were indicated with an asterisk. The entry numbers of these sequences in NCBI database were included in brackets. (C) Yeast two-hybrid analyses of the interaction between TrCYC8 and TrTUP1. Serial dilutions of yeast transformant cells harboring the indicated plasmids were spotted on double dropout medium (DDO, SD/–Leu/–Trp) as a control culture condition with no selective pressure for bait and prey interaction. The same dilutions were simultaneously spotted on quadruple dropout medium (QDO, SD/–Ade/–His/–Leu/–Trp) containing 100 ng/mL AbA with selective pressure for bait and prey interaction. The incubation was kept at 30°C for 3 days. The pGBKT7-*p53* plus pGADT7-*T* was set as a positive control. Growth was not observed for negative control transformants containing pGBKT7 plus pGADT7-*Trtup1* and pGADT7 plus pGBKT7-*Trcyc8* on QDO plate. (D) CoIP assay was performed using TrTUP1 antibody and the precipitated proteins were resolved by SDS-PAGE followed by silver staining.

To ask whether the CYC8/TUP1 complex is present in *T*. *reesei*, we searched the *T*. *reesei* genome using *S*. *cerevisiae* CYC8 (ScCYC8) or TUP1 (ScTUP1) as a query and retrieved two corresponding genes, Tr_102616 and Tr_121940, which hereafter were named *Trcyc8* and *Trtup1*, respectively. It has been well established that the ScCYC8/TUP1 complex is composed of 4 TUP1 molecules and 1 CYC8 molecule with the CYC8 tandem tetratricopeptide repeats (TPR) superhelix accommodating the TUP1 N-terminal tetramer [38, 39]. Although TrTUP1 and TrCYC8 were only 44% and 38% identical with ScTUP1 and ScCYC8 over the primary amino acid sequences (S1 and S2 Figs), domain analysis revealed the existence of 10 TPR at N terminus of TrCYC8 (43~395 aa) and 7 copies of WD40 repeats (296~605 aa) at the C-terminal portion of TrTUP1, respectively (Fig 1A). Both regions displayed a relatively higher sequence identity (57% for TPR and 60% for WD40) with their counterparts in *S*. *cerevisiae* homologs compared with other protein portions. Further phylogenetic analysis revealed that CYC8 and TUP1 sequence orthologs with similar domain structures were widely distributed among filamentous ascomycete fungi including a few well-known cellulolytic *Trichoderma*, *Penicillium*, *Fusarium*, *Neurospora*, and *Aspergillus* strains (Fig 1B), but none of these putative CYC8/TUP1 homologs have been assessed regarding their functional involvement in plant cell wall degradation.

To further verify the interaction between TrCYC8 and TrTUP1 in *T*. *reesei*, yeast two-hybrid (Y2H) and co-immunoprecipitation (CoIP) assays were performed. While Y2H results showed that TrCYC8 directly interacted with TrTUP1 (Fig 1C), CoIP assay using TrTUP1 antibody followed by mass spectrometry analysis demonstrated that TrCYC8 was readily immunoprecipitated by TrTUP1 (Fig 1D and S1 and S2 Data). Altogether, these results indicate that an evolutionarily conserved CYC8/TUP1 exists in *T*. *reesei* which may be similarly involved in gene expression regulation.

### Knockdown of *Trcyc8* or *Trtup1* compromises vegetative growth and conidiation

**Fig 2:**
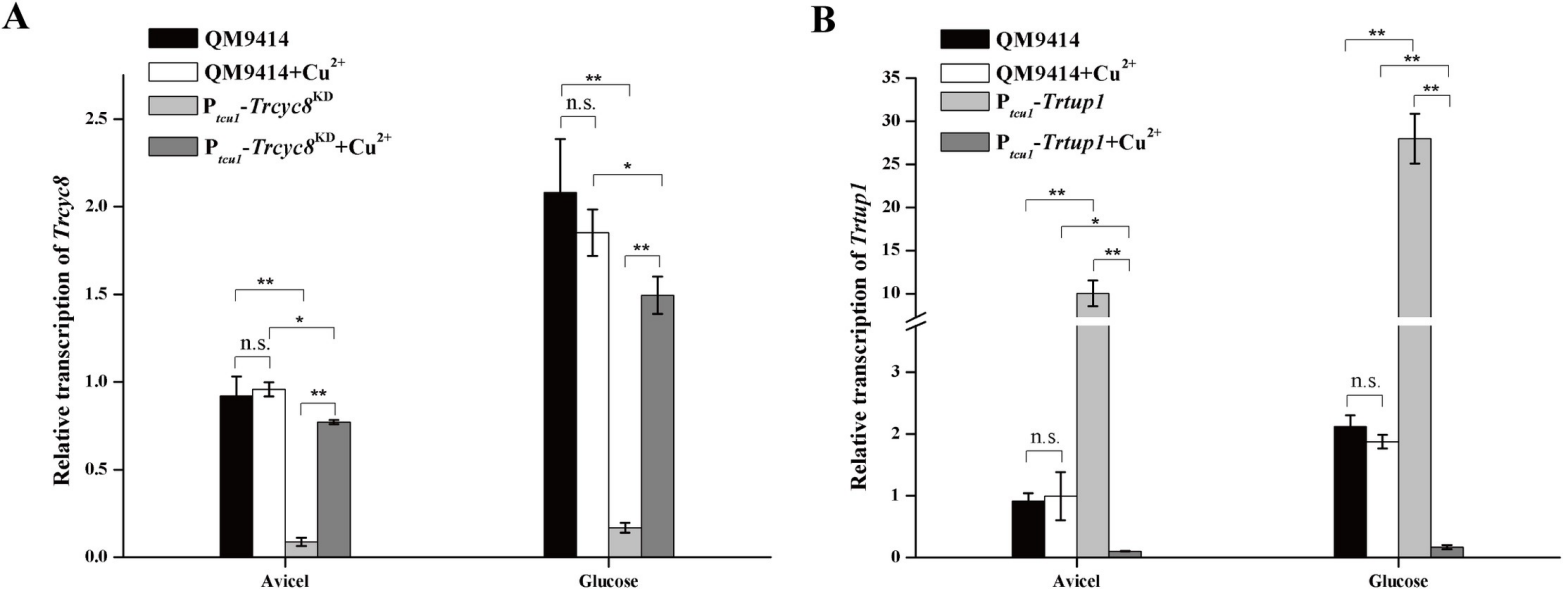
Knockdown of *Trcyc8* and *Trtup1* by conditional repression. (A) Quantitative RT-PCR analysis of the relative transcription of *Trcyc8* in P_*tcu1*_-*Trcyc8*^KD^ and QM9414 strains cultured on 1% (w/v) Avicel or glucose with or without copper. (B) Relative transcription of *Trtup1* in P_*tcu1*_-*Trtup1* and QM9414 strains cultured on 1% (w/v) Avicel or glucose with or without copper. Values are the mean of three biological replicates. Error bars are the SD from these replicates. No significant differences (*t*-test, *P*>0.05, n.s.) were observed for the transcription of *Trcyc8* or *Trtup1* in QM9414 cultured with or without copper. Significant differences (*t*-test, *P<0.05, **P<0.01) were observed for the transcription of *Trcyc8* or *Trtup1* between QM9414 and the respective recombinant strain wherein expression of *Trcyc8* or *Trtup1* was repressed without (P_*tcu1*_-*Trcyc8*^KD^) or with (P_*tcu1*_-*Trtup1*) copper under Avicel or glucose growth condition. While significant overexpression of *Trtup1* was seen between QM9414 and P_*tcu1*_-*Trtup1* cultured without copper, a slight but significant difference (*t*-test, *P<0.05) in *Trcyc8* mRNA was detected between QM9414 and P_*tcu1*_-*Trcyc8*^KD^ with copper. Significant differences (*t*-test, **P<0.01) were also observed for the transcription of *Trcyc8* or *Trtup1* in the respective recombinant strain when it was cultured with or without copper under Avicel or glucose growth condition.

To investigate the *in vivo* function of TrTUP1 and TrCYC8, constructions of the respective null mutant were first tried by replacing the coding region with the orotidine 5’-phosphate-decarboxylase gene *pyr4* in the QM9414-Δ*pyr4* strain via classical homologous recombination, but without success. Promoter substitution and RNAi strategies were then applied to achieve a copper-controlled expression of *Trtup1* and *Trcyc8*, resulting in P_*tcu1*_-*Trtup1* and P_*tcu1*_-*Trcyc8*^KD^ strains, respectively (S3 Fig) [40, 41]. As shown in Fig 2A, whereas *Trcyc8* transcripts were only slightly decreased (up to 20%) in non-repressed P_*tcu1*_-*Trcyc8*^KD^ with copper being added in the media, *Trcyc8* expression were dramatically reduced to a level about 10% of that in QM9414 in the absence of copper regardless of whether glucose or Avicel was used as the sole carbon source. Conversely, the mRNA level of *Trtup1* in P_*tcu1*_-*Trtup1* significantly decreased in the presence of copper while *Trtup1* was overexpressed without exogenously adding copper (Fig 2B).

**Fig 3:**
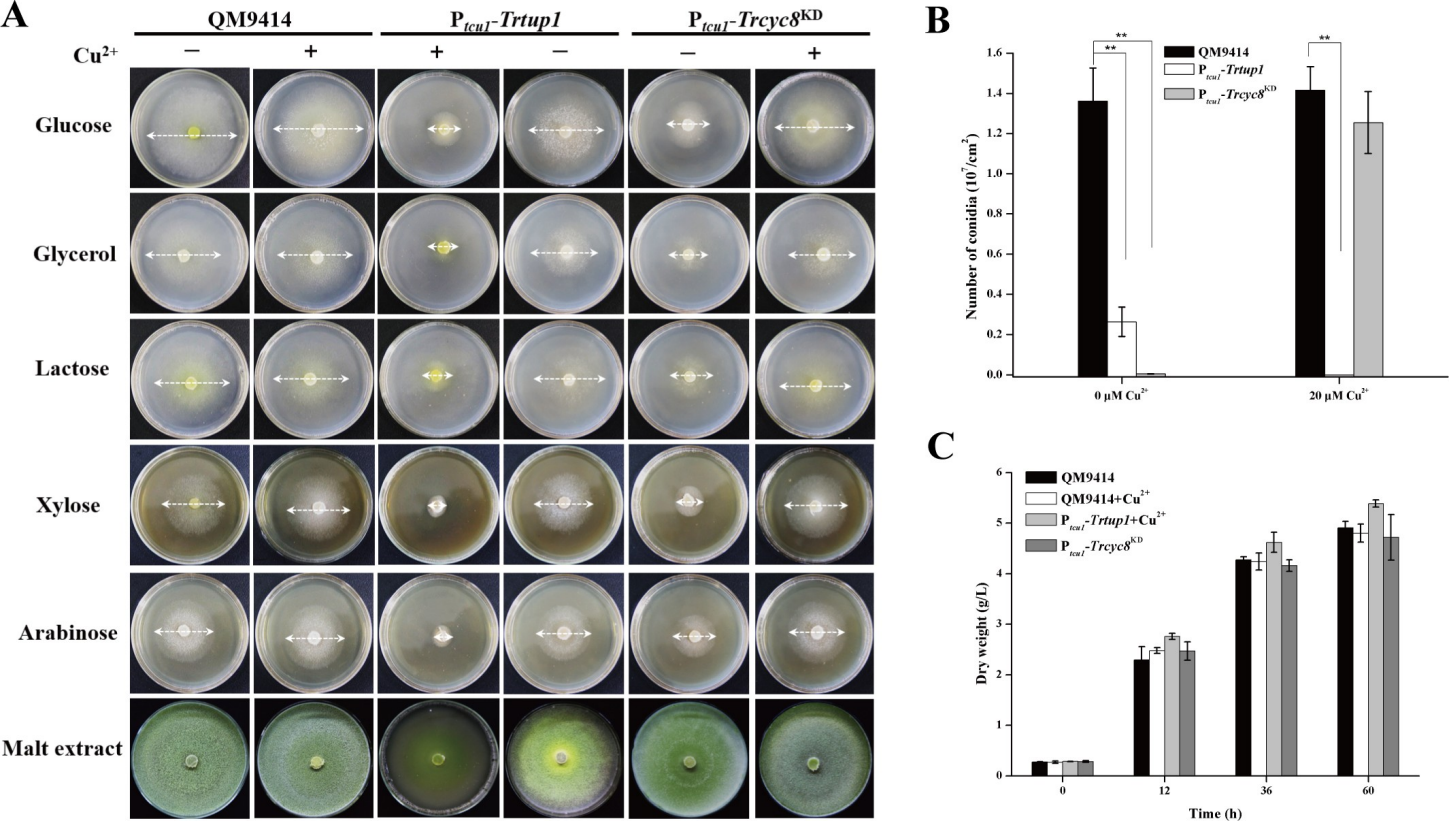
Knockdown of *Trcyc8* or *Trtup1* compromised *T*. *reesei* growth and conidiation on agar plates but not in liquid media. (A) Growth of the QM9414, P_*tcu1*_-*Trcyc8*^KD^, P_*tcu1*_-*Trtup1* strains on agar plates with various carbon sources at a final concentration of 1% (w/v) at 30°C for 3 days or on malt extract agar for 5 days. (B) Quantification of conidia formation by QM9414, P_*tcu1*_-*Trcyc8*^KD^, and P_*tcu1*_-*Trtup1* as shown in (A). Significant differences (*t*-test, **P<0.01) were observed in conidia production between QM9414 and the knock-down strains with repressed *Trtup1* and *Trcyc8*. Significant difference (*t*-test, **P<0.01) was also observed between QM9414 and P_*tcu1*_-*Trtup1* with overexpressed *Trtup1*. (C) Biomass accumulation of the P_*tcu1*_-*Trcyc8*^KD^, P_*tcu1*_-*Trtup1* and QM9414 strains in MA liquid culture with 1% glucose as the sole carbon source. Mycelia dry weight was determined for biomass quantification. No statistical difference (*t*-test, P>0.05) was observed for the growth of these strains.

Further analysis of the respective knock-down strain showed that transcriptional repression of *Trcyc8* or *Trtup1* compromised vegetative growth on plate with various tested carbon sources and conidiation on malt extract agar plate (Fig 3A and 3B), phenotypes that are consistent with those found in *A*. *nidulans* and *A*. *niger* [42, 43]. *Trtup1* overexpression in the absence of copper also resulted in slowed growth and reduced conidiation on solid media (Fig 3A and 3B). Unlike growth on plate, there was hardly any difference in biomass accumulation between the knockdown mutants and QM9414 in liquid culture with glucose as carbon source (Fig 3C).

### *Trcyc8* or *Trtup1* repression abolishes cellulase gene expression

**Fig 4:**
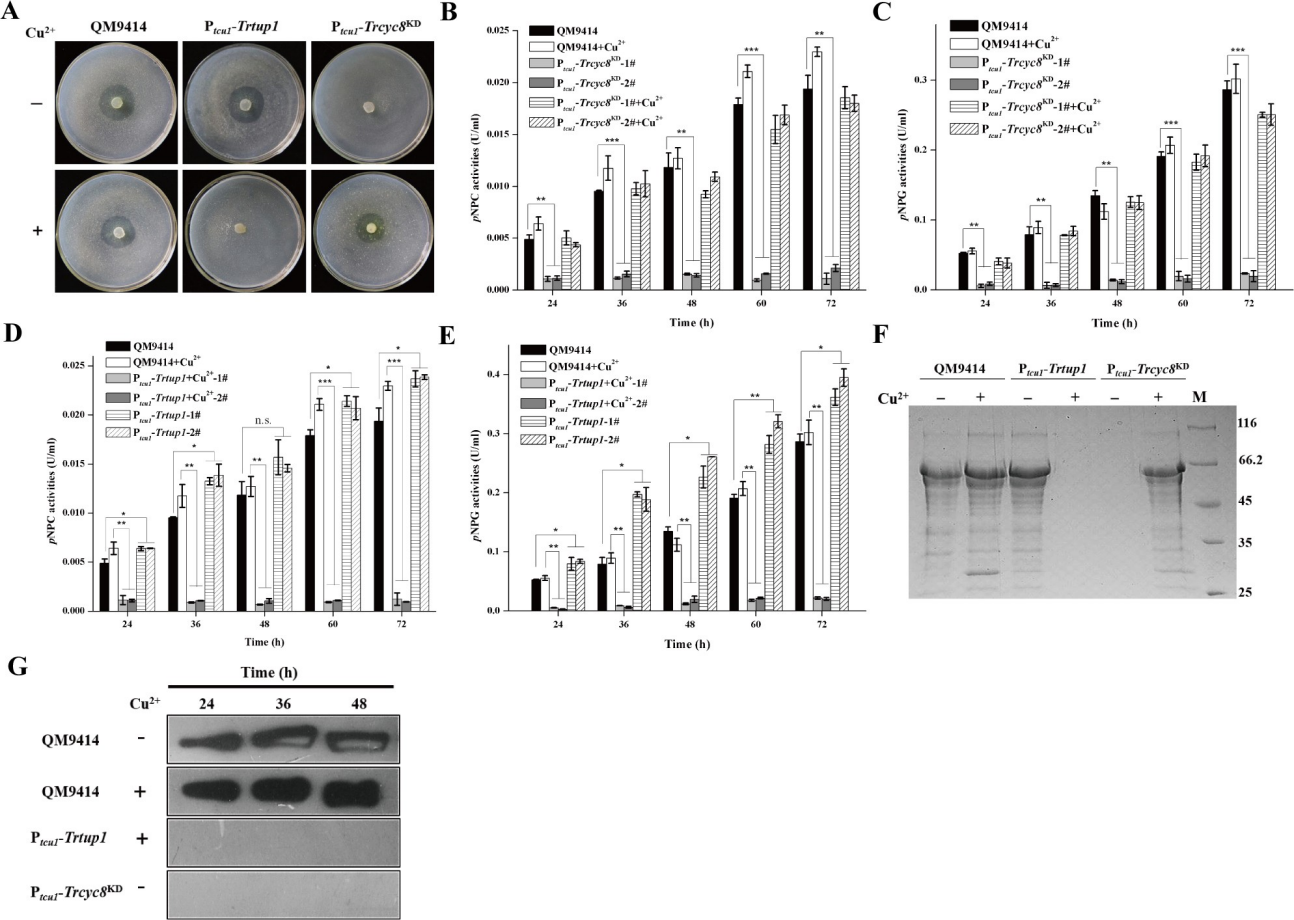
Knockdown of *Trcyc8* and *Trtup1* abolished cellulase production. (A) Hydrolytic halo formed by the P_*tcu1*_-*Trcyc8*^KD^, P_*tcu1*_-*Trtup1* and QM9414 strains on plate covered with a layer of 0.4% (w/v) ground Avicel with or without copper. (B-E) Extracellular *p*NPC and *p*NPG hydrolytic activities of the culture supernatant from QM9414 and two independent transformants of P_*tcu1*_-*Trcyc8*^KD^ (B-C) or P_*tcu1*_-*Trtup1* (D-E) strains cultured on 1% (w/v) Avicel with or without copper for the indicated time periods. Values are the mean of three biological replicates. Error bars are the SD from these replicates. Significant differences (*t*-test, *P<0.05, **P<0.01, ***P<0.001) were detected for the extracellular *p*NPCase and *p*NPGase activities between QM9414 and two independent transformants of P_*tcu1*_-*Trcyc8*^KD^ or P_*tcu1*_-*Trtup1* with or without copper for the indicated time points after induction. (F) SDS-PAGE analysis of the culture supernatant from the P_*tcu1*_-*Trcyc8*^KD^, P_*tcu1*_-*Trtup1* and QM9414 strains after induction 72 h on 1% (w/v) Avicel. Equal amounts of Avicel culture at the indicated time points after inoculation of pre-cultured mycelia were taken for measuring extracellular protein. (G) Western blot analysis of extracellular CEL7A(CBH1) in the culture supernatant of QM9414 and the knock-down strains with repressed *Trtup1* and *Trcyc8* after induction on Avicel at indicated time.

To check the cellulolytic effect of *Trtup1* or *Trcyc8* repression, QM9414, P_*tcu1*_-*Trcyc8*^KD^ and P_*tcu1*_-*Trtup1* were simultaneously inoculated and incubated on solid medium covered with a top agar layer containing ground Avicel (0.4%, w/v) with or without copper (Fig 4A). In contrast with the control and non-repressed strains, knockdown of *Trcyc8* and *Trtup1* completely eliminated the formation of hydrolytic haloes. Analysis of the extracellular hydrolytic activities towards various substrates using the respective culture supernatant on cellulose with or without copper revealed that *Trcyc8* or *Trtup1* repression abolished extracellular *p*NPC and *p*NPG hydrolytic activities, while *Trtup1* overexpression resulted in an approximate 20~100% increase in extracellular cellobiohydrolase and β-glucosidase activities (Fig 4B–4E). SDS-PAGE and western blot analyses of the secreted enzyme components confirmed that hardly any cellulase component could be detected upon cellulose induction when *Trtup1* or *Trcyc8* expression was turned down (Fig 4F and 4G). *Trcyc8* or *Trtup1* repression also resulted in an up to 80% reduction in the induced expression of xylanases upon xylan (S4 Fig).

**Fig 5:**
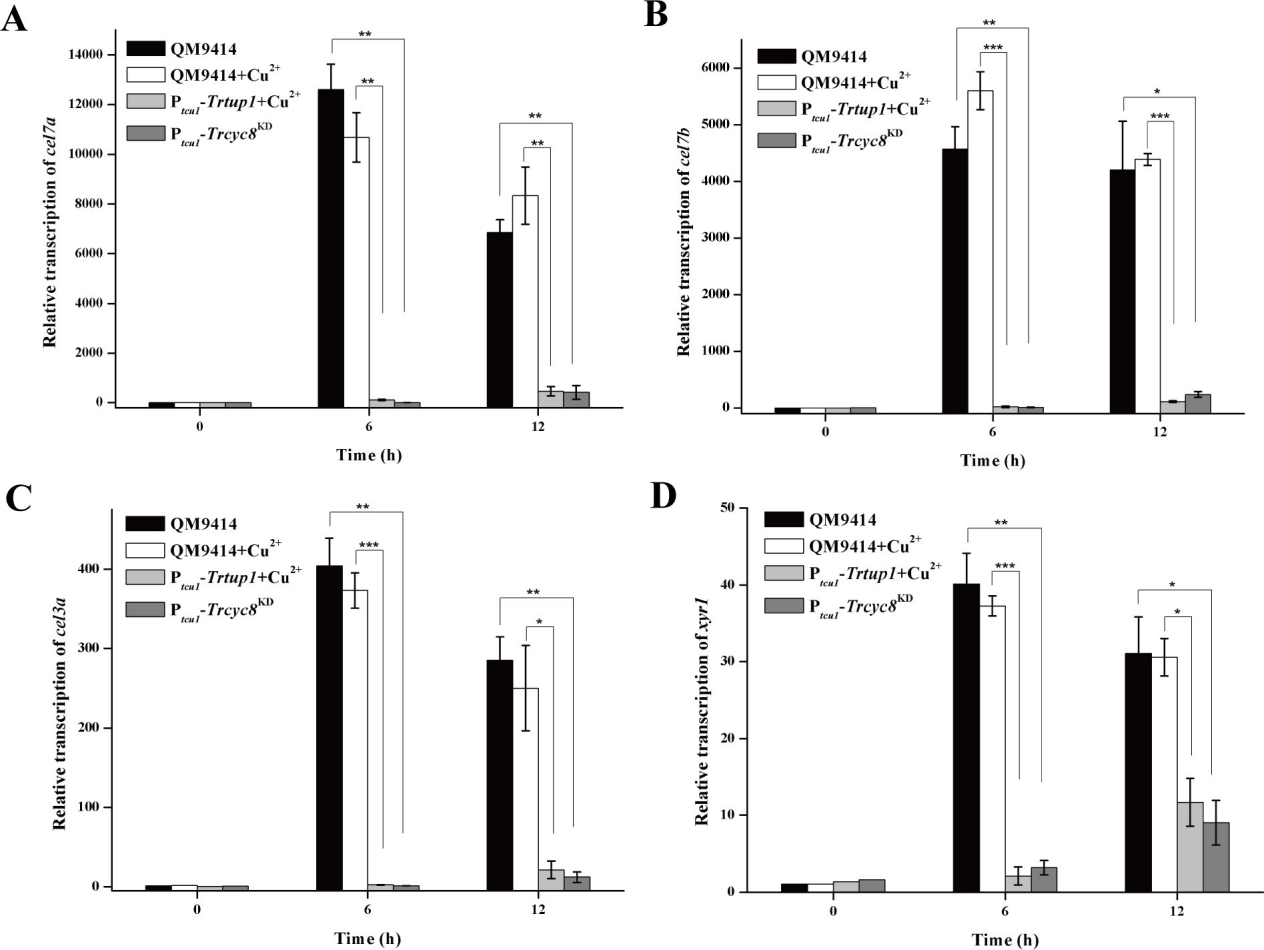
*Trcyc8* or *Trtup1* repression resulted in a significant decrease in the transcription of cellulase gene and *xyr1* transcription. Transcription of *cel7a* (A), *cel7b* (B), *cel3a* (C) and *xyr1* (D) was analyzed by quantitative RT-PCR after induction on 1% (w/v) Avicel for the indicated time periods. The expression level of the *actin* gene was used as a reference gene for normalization in all samples. Values are the mean of three biological replicates. Error bars are the SD from these replicates. Significant differences (*t*-test, *P<0.05, **P<0.01, ***P<0.001) were detected for the transcription of *cel7a*, *cel7b*, *cel3a*, and *xyr1* between QM9414 and *Trcyc8*^KD^ or *Trtup1* repressed cells.

Further examination of endogenous *cel7a* (*cbh1*), *cel7b* (*egl1*), *cel3a* (*bgl1*), and *xyr1* mRNA levels by qRT-PCR demonstrated that the drastically reduced cellulase activities as observed in the repressed strains resulted from a down-regulation in the steady state transcripts of these cellulase genes as well as the *xyr1* gene (Fig 5). Altogether, the data indicate that TrCYC8/TUP1 plays an important role in mediating the induced cellulase gene expression.

### TrTUP1 is recruited to cellulase gene promoters that relies on XYR1

**Fig 6:**
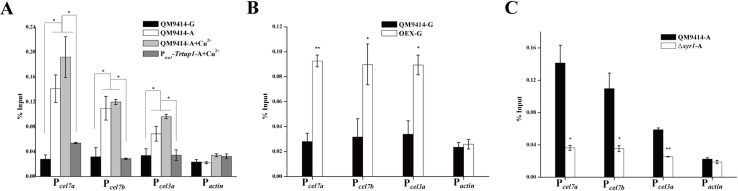
TrTUP1 was recruited to cellulase gene promoters upon induction in an XYR1-dependent manner. (A) ChIP assay for TrTUP1 binding to cellulase gene promoters in the QM9414 and P_*tcu1*_-*Trtup1* strains cultured on Avicel or glucose with or without copper. (B) ChIP assay for TrTUP1 binding to cellulase gene promoters in the QM9414 and OEX strains cultured on glucose in the absence of copper allowing for XYR1 overexpression. (C) ChIP assay for TrTUP1 binding to cellulase gene promoters in the QM9414 and Δ*xyr1* strains cultured on Avicel. The actin promoter was used as a negative control. G stands for glucose and A stands for Avicel. Significant differences (*t*-test, *P<0.05, **P<0.01) were detected for TrTUP1 binding when XYR1 was induced on Avicel or overexpressed on glucose compared to when XYR1 was not expressed.

To test whether TrCYC8/TUP1 is recruited to the cellulase gene promoter and directly involved in activating cellulase gene expression, chromatin immunoprecipitation (ChIP) followed by quantitative PCR (ChIP-qPCR) on selected promoter regions was performed using TrTUP1 antibody to determine TrTUP1 occupancy on cellulase gene promoters. As shown in Fig 6A, TrTUP1 was significantly enriched on *cel7a*, *cel7b*, and *cel3a* promoters in QM9414 cultured on Avicel regardless of the presence or absence of copper. No significant enrichment of TrTUP1 was detected on the same promoters in P_*tcu1*_-*Trtup1* cultured in the presence of copper wherein the *TrTup1* expression was repressed as well as on the *actin* promoter (Fig 6A). Since the TrCYC8/TUP1 complex does not have the ability to bind DNA sequences by itself, its recruitment to target gene promoters strictly depends on specific DNA binding proteins [44]. To further ask whether TrTUP1 recruitment to cellulase gene promoters depends on XYR1, ChIP assays were performed either in the Δ*xyr1* strain cultured on Avicel or in QM9414 overexpressing XYR1 (OEX) cultured on glucose. XYR1 overexpression has been reported to lead to a high-level cellulase gene expression even under repressing condition[34]. In accordance with this, TrTUP1 binding was found to occur even when cultured with glucose as the sole carbon source wherein XYR1 was overexpressed simultaneously (Fig 6B). In contrast, no significant TrTUP1 binding to cellulase gene promoters was observed in the absence of XYR1 under cellulase inducing conditions (Fig 6C). Taken together, these results indicate that TrTUP1 recruitment to cellulase gene promoters specifically depends on XYR1.

### TrTUP1 and TrCYC8 are required for efficient binding of XYR1 to cellulase gene promoters *in vivo*

**Fig 7:**
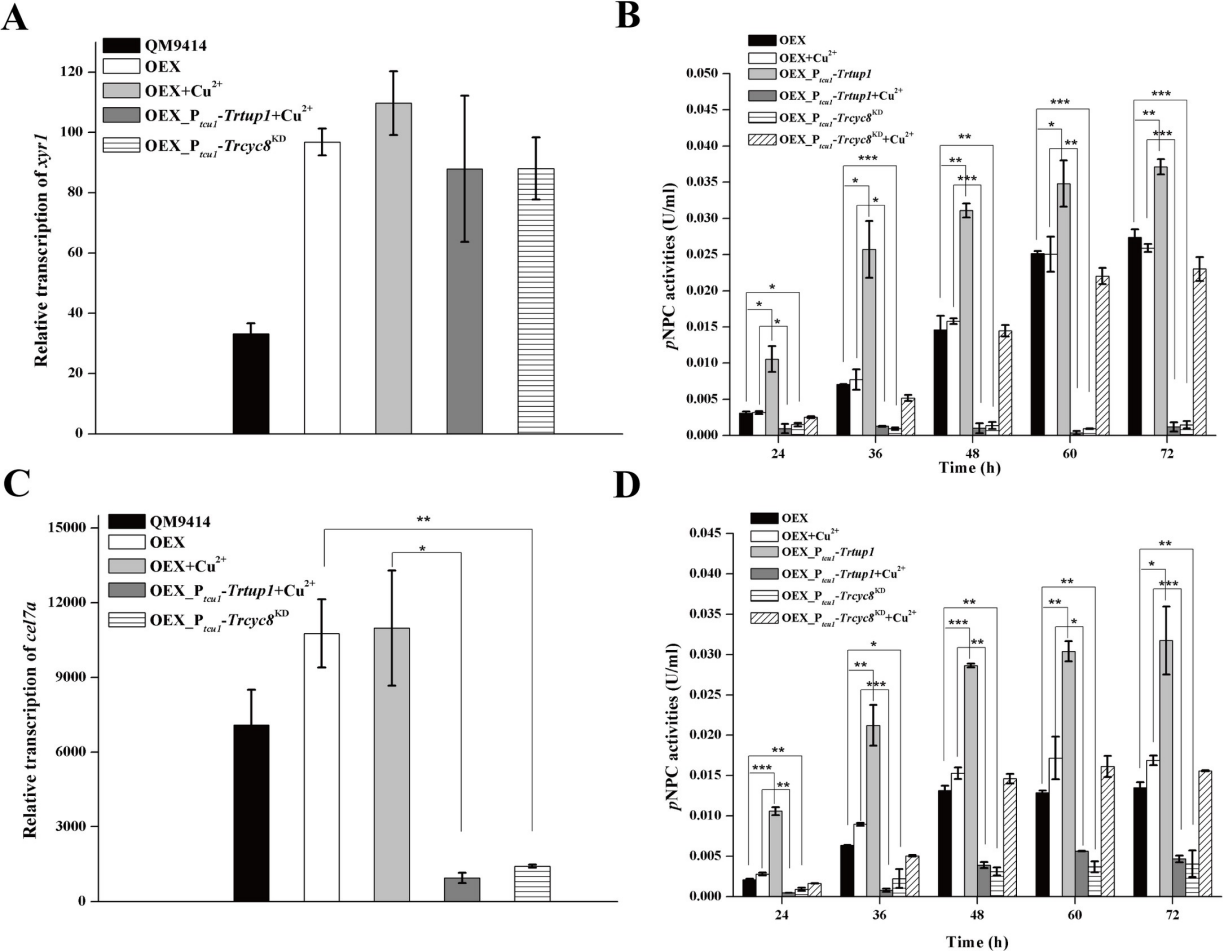
XYR1 overexpression failed to rescue the defective cellulase production in P_*tcu1*_-*Trcyc8*^*KD*^ or P_*tcu1*_-*Trtup1*. (A) Quantitative RT-PCR analyses of transcription of *xyr1* in QM9414, OEX, OEX_P_*tcu1*_-*Trtup1*, and OEX_P_*tcu1*_-*Trcyc8*^KD^ strains cultured on Avicel with or without copper for 12 h. (B) Extracellular *p*NPC hydrolytic activities of the culture supernatant from the OEX, OEX_P_*tcu1*_-*Trcyc8*^KD^ and OEX_P_*tcu1*_-*Trtup1* strains cultured on Avicel with or without copper. (C) Relative transcription of *cel7a* in QM9414, OEX, OEX_P_*tcu1*_-*Trtup1* and OEX_P_*tcu1*_-*Trcyc8*^KD^ strains cultured on Avicel with or without copper for 12 h. (D) Extracellular *p*NPC hydrolytic activities of the culture supernatant from the OEX, OEX_P_*tcu1*_-*Trcyc8*^KD^ and OEX_P_*tcu1*_-*Trtup1* strains cultured on glucose with or without copper. Significant differences (*t*-test, *P<0.05, **P<0.01, ***P<0.001) were detected for *cel7a* transcription and *p*NPCase activity between OEX and OEX_P_*tcu1*_-*Trtup1* or OEX_P_*tcu1*_-*Trcyc8*^KD^ wherein *Trtup1* or *Trcyc8* was repressed. Significant differences (*t*-test, *P<0.05, **P<0.01, ***P<0.001) were also detected for *p*NPCase activity between OEX and OEX_P_*tcu1*_-*Trtup1* wherein *Trtup1* was overexpressed.

Considering that *xyr1* transcription was severely down-regulated with *Trcyc8* or *Trtup1* repression, we asked whether restoring XYR1 expression is able to rescue the activation defects observed with *Trtup1* or *Trcyc8* repressed cells. Recombinant strains that simultaneously overexpressed *xyr1* under the control of the *cdna1* promoter in P_*tcu1*_-*Trtup1* (OEX_P_*tcu1*_-*Trtup1*) or P_*tcu1*_-*Trcyc8*^KD^ (OEX_P_*tcu1*_-*Trcyc8*^KD^) were thus constructed (S5 Fig). Comparable *xyr1* mRNA levels were observed in OEX_P_*tcu1*_-*Trtup1* or OEX_P_*tcu1*_-*Trcyc8*^KD^ strain and the OEX strain, which were up to 3-fold higher than those in QM9414 cultured on Avicel (Fig 7A). Extracellular *p*NPC hydrolytic activity and qRT-PCR analysis of the induced *cel7a* transcription revealed that XYR1 overexpression failed to rescue the induction defect resultant from the knockdown of *Trcyc8* or *Trtup1* (Fig 7B and 7C). On the other hand, simultaneous overexpression of TrTUP1 and XYR1 in OEX_P_*tcu1*_-*Trtup1* without copper resulted in a significant increase in cellulase production not only on Avicel but also on glucose (up to 120%) compared with the OEX strain at almost all time points assayed after inoculation. Again, repression of *Trtup1* or *Trcyc8* crippled the glucose-blind cellulase production resultant from XYR1 overexpression (Fig 7D).

**Fig 8:**
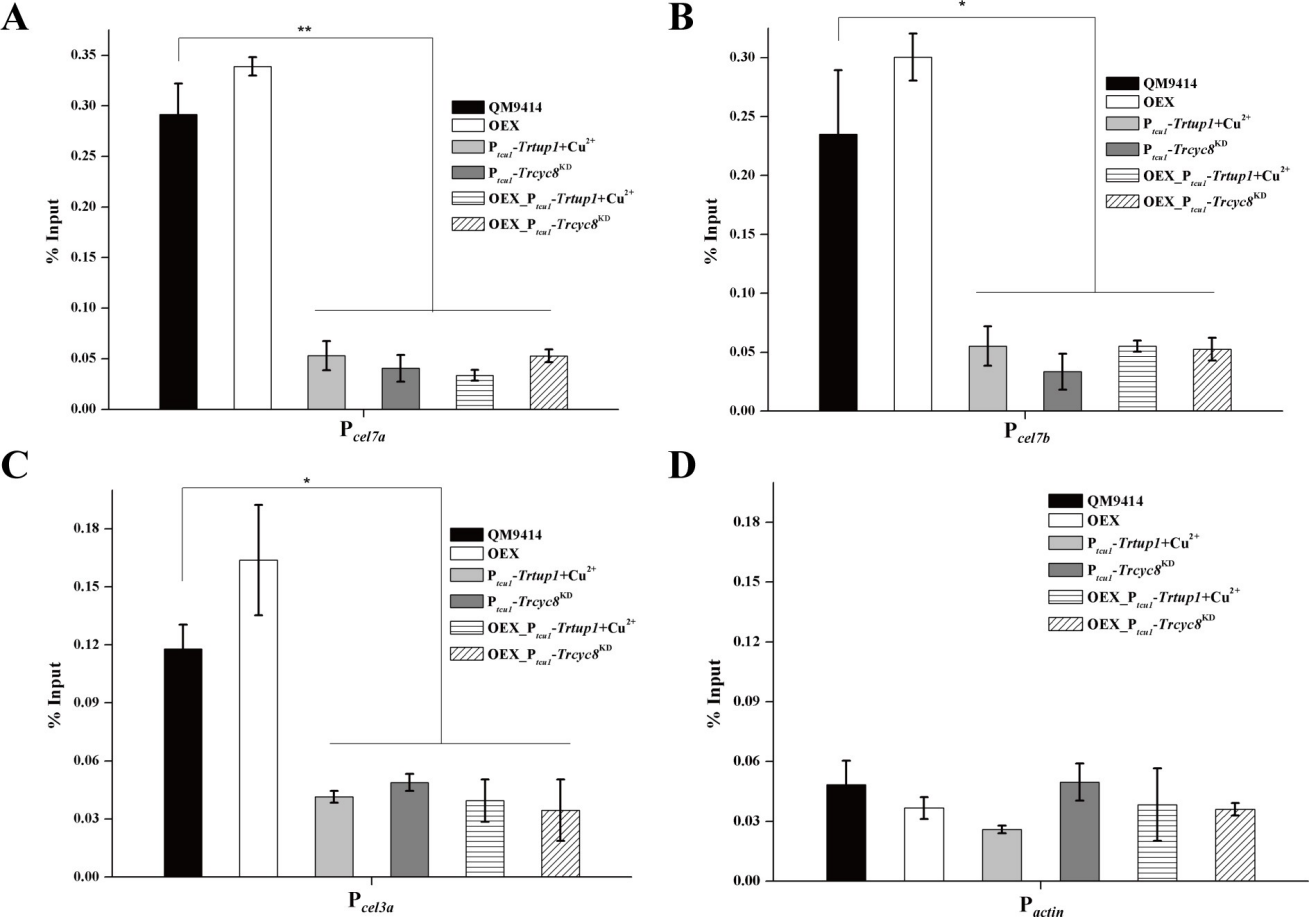
Efficient binding of XYR1 to cellulase genes in vivo relied on TrTUP1. (A-D) ChIP assay for XYR1 binding to *cel7a* (A), *cel7b* (B), *cel3a* (C) and *actin* (D) promoters in the QM9414, OEX, P_*tcu1*_-*Trcyc8*^KD^, P_*tcu1*_-*Trtup1*, OEX_P_*tcu1*_-*Trtup1*, and OEX_P_*tcu1*_-*Trcyc8*^KD^ strains cultured on 1% (w/v) Avicel with or without copper for 12 h. Significant differences (*t*-test, *P<0.05, **P<0.01) were detected for XYR1 binding to *cel7a*, *cel7b* and *cel3a* promoters between the control strain and knock-down strains wherein *Trtup1* or *Trcyc8* was repressed. No significant differences (*t*-test, P>0.05, n.s.) were detected for XYR1 binding to the *actin* promoter.

Given that XYR1 overexpression failed to rescue the general expression impairment, we next tested using ChIP analysis whether binding of XYR1 to its target promoters is impaired by *Trcyc8* or *Trtup1* repression (Fig 8). Results showed that binding to cellulase gene promoters of endogenous XYR1 was greatly reduced in the *Trtup1* or *Trcyc8* repressed cells compared to the binding in QM9414 cultured on Avicel. While XYR1 overexpression increased its binding to cellulase gene promoters in QM9414, it did not rescue its defective binding with repressed *Trtup1* or *Trcyc8*. Thus, TrCYC8/TUP1 seems to be critical for efficient binding of XYR1 on cellulase gene promoters.

### *Trcyc8* or *Trtup1* repression interferes with the induced loss of nucleosomes in cellulase gene promoters

**Fig 9:**
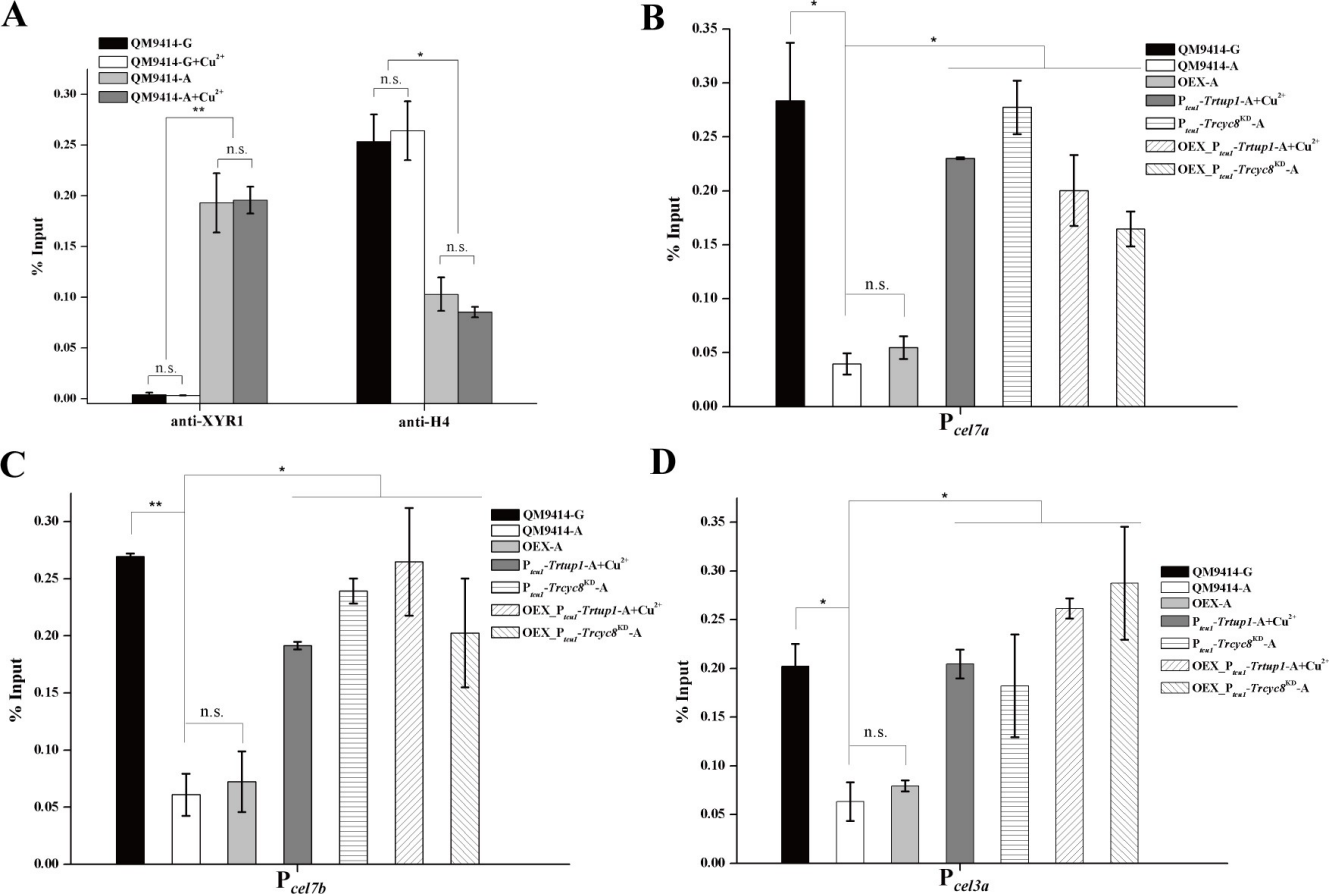
The effect of *Trcyc8* or *Trtup1* repression on the association of histone H4 with cellulase gene promoters upon cellulose induction. (A) Analysis of XYR1 binding and H4 association with the *cel7a* promoter in the control strain QM9414 cultured on 1% (w/v) Avicel or glucose with or without copper for 12 h using ChIP followed quantitative RT-PCR. (B-D) ChIP analysis of H4 association with the *cel7a* (B), *cel7b* (C) and *cel3a* (D) promoters in the QM9414, P_*tcu1*_-*Trcyc8*^KD^, P_*tcu1*_-*Trtup1*, OEX, OEX_P_*tcu1*_-*Trtup1* and OEX_P_*tcu1*_-*Trcyc8*^KD^ strains cultured on 1% (w/v) Avicel or glucose with or without copper for 12 h. G stands for glucose and A stands for Avicel. Significant differences (*t*-test, *P<0.05, **P<0.01) were detected for H4 occupation on the *cel7a*, *cel7b* and *cel3a* promoters between the control strain and knock-down strains wherein *Trtup1* or *Trcyc8* was repressed.

One key aspect of CYC8/TUP1 function in gene repression or activation is linked to alteration of the local chromatin structure by recruiting chromatin modifying or remodeling complexes [13, 18, 19]. To ask whether TrCYC8/TUP1 recruitment is associated with chromatin status change in cellulase gene promoters, loss of nucleosome components from the indicated promoter regions was tracked by ChIP to assay the loss of histone H4. Upon cellulose induction, an increased binding of XYR1 along with a significant loss of histone H4 to the *cel7a* gene promoter was observed in QM9414 (Fig 9A). Similar to TrTUP1 and XYR1, histone H4 association with the *cel7a* promoter was not influenced by the presence or absence of copper in the control strain. In contrast, H4 occupancy appeared to be persistent on cellulase gene promoters in *Trtup1* or *Trcyc8* repressed cells even with cellulose induction (Fig 9B–9D). Again, XYR1 overexpression had no effect on repositioning or removing H4 at these promoter regions with repressed *Trtup1* or *Trcyc8* (Fig 9B–9D). In all these data suggest that TrCYC8/TUP1 is involved in remodeling nucleosomes positioned in cellulase gene promoters to activate their transcription although more precise regulatory mechanism needs to be investigated.

## Discussion

The results of this study show that a TrCYC8/TUP1 complex exists in *T*. *reesei*, and is required for high-level activation of cellulase genes *in vivo*. Similar to functions in other filamentous fungi, repression of *Trcyc8* or *Trtup1* results in gross defects in vegetative growth and asexual spore production in *T*. *reesei* [42, 43]. Unlike the carbon catabolite repressor CRE1, the TrCYC8/TUP1 complex is critical for the induced cellulase gene expression. ChIP analyses reveal that TrTUP1 is recruited to cellulase gene promoters in an XYR1-dependent manner, whereas at the same time *Trtup1-*repressed cells exhibits diminished recruitment of XYR1. Thus, XYR1 and TrCYC8/TUP1 are interdependent for efficient binding at cellulase gene promoters to ensure their induced expression.

As one of the first complexes to be defined as a transcriptional corepressor, the importance of this evolutionary conserved complex is not only well documented in budding yeast to mediate repression of diverse sets of genes, but also highlighted by the presence of homologs in *Schizosaccharomyces pombe*, *Caenorhabditis elegans* and the evolutionary relatives in metazoans, the Gro/TLE family of corepressors [14]. In the yeast *S*. *cerevisiae*, it has been exemplified that CYC8/TUP1 is tethered to glucose-repressible promoters (such as *suc2* or *gal1*) via the transcriptional repressor MIG1 to mediate CCR [45]. Nonetheless, neither *mig1* nor *tup1* deletion compromises activation of transcription from those promoters following derepression [12, 18]. As a MIG1 ortholog in *T*. *reesei*, CRE1 is similarly established as the main transcription factor mediating CCR, and the deletion of *cre1* contributes to the improved cellulase production [33]. It should be pointed out that the parental strain used in our study, QM9414, is a mutagenized derivative of QM6a. Although there has been no report on any mutation occurring in CRE1 in QM9414, one might assume that other genome aberrations could directly or indirectly affect the cellulase gene regulatory system involving TrCYC8/TUP1. Nonetheless, the impaired cellulase gene expression with repressed *Trcyc8* or *Trtup1* in this study implicates that the complex may act in a different mode from that of CRE1 in controlling the cellulolytic response in *T*. *reesei* although possible interactions between CRE1 and TrCYC8/TUP1 can not be excluded.

Interestingly, the CYC8/TUP1 complex has been also reported to function in gene activation. In these reported situations, the already bound CYC8/TUP1 is converted from a corepressor to a coactivator under inducing conditions. The followed TUP1-dependent recruitment of SAGA histone acetylase cofactor complex and SWI/SNF ATP-dependent chromatin remodeling complex just appears to be required primarily to counteract CYC8/TUP1 repression as deletion of TUP1 does not reduce transcription of these genes [18, 19, 46]. Our observations that TrCYC8/TUP1 is absolutely required for induced cellulase gene expression and is only recruited to cellulase promoters upon induction indicate that a different mode of activation by TrCYC8/TUP1 is adopted. The scenario described here is more like the situation described for GCN4, a transcriptional activator of amino acid biosynthetic genes in yeast [23]. ScCYC8 and, to a lesser extent, ScTUP1 are required for high-level activation of GCN4 target genes *in vivo* by achieving an interdependent binding to *arg1* and *arg4* [23]. Nevertheless, there has been no report on CYC8/TUP1 function in gene activation in filamentous fungi although RCM1/RCO1, the CYC8/TUP1 homolog in *Neurospora crassa*, has been reported to mediate specific repression of the *frequency* gene [47, 48].

Considering the critical role of XYR1 in the induced cellulase gene expression, it is reasonable to attribute the observed defect in transcriptional activation of cellulase genes with repressed *Trtup1* or *Trcyc8* to the impaired XYR1 binding to its target genes. This decrease in promoter occupancy by XYR1 cannot be accounted for by reduced expression of XYR1 since XYR1 overexpression failed to rescue the induction defect in *Trtup1* or *Trcyc8* knockdown cells. Although the exact mechanism is not clear yet, a direct role for TrCYC8/TUP1 in promoting the promoter binding by XYR1 is suggested by the following observations. TrTUP1 recruitment to cellulase gene promoters depends on XYR1. Moreover, simultaneous overexpression of XYR1 and TrTUP1 significantly enhances cellulase gene expression. Possibility thus exists that the recruited TrCYC8/TUP1 complex act by itself or synergizing with other cofactors including the SAGA histone acetylase cofactor complex and the SWI/SNF ATP-dependent chromatin remodeling complex to modify the local chromatin structure to increase accessibility of XYR1 to its binding sites. Another possibility is that the recruited TrCYC8/TUP1 somehow facilitates the formation of a transcriptional active complex conductive from XYR1 to the PIC wherein XYR1 binding is enhanced. Apart from promoting XYR1 binding, the recruited TrCYC8/TUP1 may also actively participate in the activation process. In this respect, it has been reported that CYC8 and, to a lesser extent, TUP1 are required for activation of a plasma membrane ferric reductase gene *fre2* by AFT1 in yeast [20]. Notwithstanding the slightly reduced AFT1 binding at *fre2* without CYC8, activation by a LexA-AFT1 fusion from LexA binding sites was strongly CYC8 dependent, pointing to a role for CYC8 in the activation function of AFT1 rather than in AFT1 promoter binding [20]. It would thus be interesting to test in the future whether artificial recruitment of XYR1 would bypass the requirement of TrCYC8/TUP1 for cellulase gene activation. Regardless of this, given the multiple protein interaction sites on CYC8/TUP1 and its significant potential of being incorporated in different complexes [15–19], it is reasonable to anticipate that the way by which CYC8/TUP1 achieves its repression may also apply to its activation. Our previous results demonstrate that XYR1 directly interacts with SWI/SNF and the Mediator tail subunit to facilitate the recruitment of the general transcription machinery including RNA Pol II to successfully initiate the transcription of these cellulase genes [49, 50]. One possibility is thus that, upon recruited by XYR1, the TrCYC8/TUP1 complex cooperates with XYR1 to achieve the efficient recruitment of the above components allowing the rapid and high-level cellulase gene activation.

As with other corepressors, the specificity of repression mediated by CYC8/TUP1 is determined by a number of sequence-specific DNA binding repressors, which recruit the complex to turn off subsets of genes. However, CYC8/TUP1 recruitment by transcriptional activators has been also demonstrated for GCN4 and AFT1 that activate amino acid biosynthetic genes and the *fre2* gene in yeast, respectively [20, 23]. In the present study, we show that TrTUP1 is recruited to cellulase gene promoter in an XYR1-dependent manner. Although repeated efforts have been made to detect a direct interaction between XYR1 and TrCYC8/TUP1 using yeast two-hybrid system, pull-down and Co-IP assays, no evidence has been uncovered to support this possibility. We presume that XYR1 recruits TrCYC8/TUP1 to cellulase gene promoters in a manner dependent on other coactivators or histone modifications that are recruited by XYR1 or TrCYC8/TUP1 initially bound with low-efficiency. Once arriving at target promoters, synergistic actions of TrCYC8/TUP1 and XYR1 enhance their interdependent binding. Future experiments will address the mechanism how this indirect recruitment is achieved.

**Fig 10:**
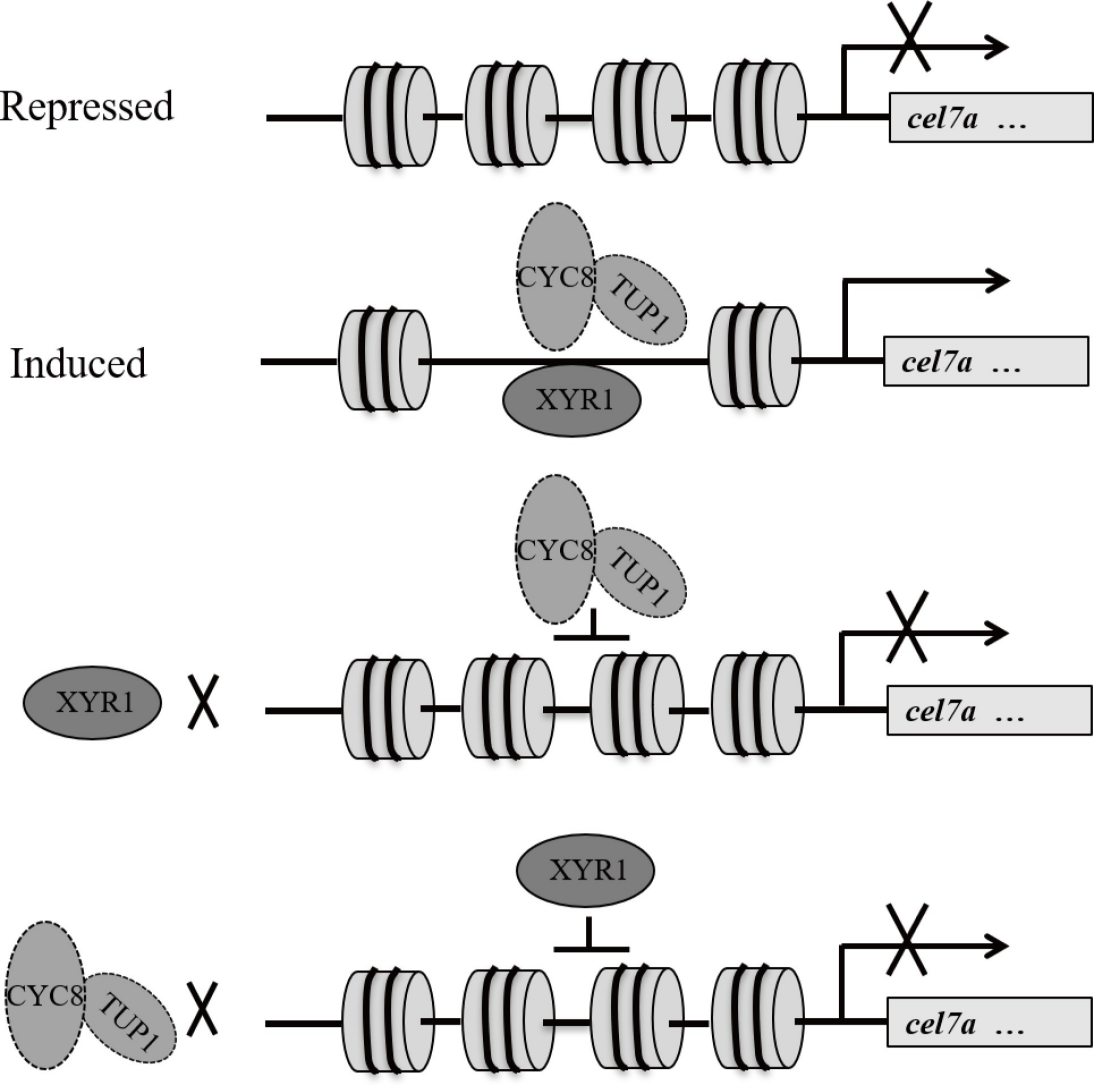
A working model for interdependent binding of TrCYC8/TUP1 and XYR1 on cellulase gene promoters to mediate their induced expression. When glucose is present, nucleosome positioning in cellulase gene promoters impedes binding of the transcription activator XYR1 as well as the general transcriptional machinery. Upon cellulose induction, interdependent binding of XYR1 and TrCYC8/TUP1 provides a synergistic mechanism to achieve efficient binding of both regulators to cellulase gene promoters. Besides promoting XYR1 binding, TrCYC8-TUP1 may also actively participate in cooperation with XYR1 in recruiting other coactivators and PIC to ensure the successful initiation of cellulase gene transcription.

Based on the presented data, a working model for TrCYC8/TUP1 mode of action during cellulase gene expression is proposed in Fig 10. Under repressing conditions, nucleosome positioning in cellulase gene promoters limits the accessibility of the involved regulatory regions to relative transcription (co)activators as well as the general transcriptional machinery. Upon cellulose induction, interdependent binding of XYR1 and TrCYC8/TUP1 provides a synergistic mechanism to achieve efficient binding of both regulators to cellulase gene promoters under inducing condition. In addition to promoting XYR1 binding, TrCYC8/TUP1 may also actively participate in cooperation with XYR1 in recruiting other coactivators and PIC to ensure the successful initiation of cellulase gene transcription. The findings also implicate an engineering strategy via overexpression of the TrCYC8/TUP1 complex to help to enhance cellulase production even over levels achieved in already existing *T*. *reesei* mutations.

## Materials and methods

### Strains, media, and culture conditions

*Escherichia coli* DH5α cells were cultured in Luria-Bertani media at 37°C and used for plasmids construction and amplification. *Saccharomyces cerevisiae* Y2H Gold (*MATa*, *ura3-52*, *his3-200*, *ade2-101*, *trp1-901*, *leu2-3*, *112*, *gal4*Δ, *gal80*Δ, LYS2::*GAL1*_*UAS*_-*Gal1*_*TATA*_-His3,*GAL2*_*UAS*_-*Gal2*_*TATA*_*-Ade2URA3*::*MEL1*_*UAS*_-*Mel1*_*TATA*_ *AUR1-C MEL1*) was used as the host for two-hybrid screen. Yeast cells were routinely cultivated at 30°C in YPD medium (1% yeast extract, 2% peptone, and 2% glucose) for 12–18 h to reach an OD_600_ of 0.6. Synthetic complete (SC) medium lacking tryptophan, leucine, histidine, and adenine (QDO, SD/–Ade/–His/–Leu/–Trp) with 100 ng ml^-1^ of Aureobasidin A (AbA) was used for cultivation of recombinant yeast transformants. *T*. *reesei* QM9414 (ATCC 26921) and QM9414-Δ*pyr4* in which the uridine trophic gene was deleted in QM9414 [51], were used throughout this work as the control and parental strain, respectively. All *T*. *reesei* strains were maintained on malt extract agar (3% malt extract, 0.5% peptone, and 1.5% agar A) supplemented with 10 mM uridine when necessary. For cellulase production and gene transcription analyses, *T*. *reesei* strains were pre-grown in 1 L Erlenmeyer flasks containing 250 ml Mandels-Andreotti (MA) medium with 1% (v/v) glycerol as carbon source on a rotary shaker (200 rpm) at 30°C for 48 under the natural light-dark cycle as previously described [52]. Mycelia were harvested by G1 sand core funnel (100~160 μm) and washed twice (3~5 min each time) with MA medium without carbon source. Equal amounts of mycelia were then transferred to fresh MA medium containing 1% (w/v) Avicel, and incubation was continued for the indicated time periods. Copper at a final concentration of 20 μM was present in the media when necessary.

### Yeast two-hybrid (Y2H)

All Y2H assays were performed according to the manufacture’s manual (Clontech, Mountain View, CA, USA). In order to analyze the interaction between TrCYC8 and TrTUP1, the full-length *Trcyc8* and *Trtup1* cDNA sequences were obtained by fusion of their respective exons amplified from *T*. *reesei* genomic DNA using overlap-extension PCR [53], and were inserted into the *Eco*RI/*Bam*HI sites of pGBKT7 and *Eco*RI/*Xho*I sites of pGADT7, respectively, resulting in pGBKT7-*Trcyc8* and pGADT7-*Trtup1*, which were subsequently co-transformed into the *S*. *cerevisiae* Y2H Gold cells. The Y2H Gold cells containing pGBKT7-*p53* and pGADT7-*T* were set as a positive control in Y2H assays.

### Protein co-immunoprecipitation (Co-IP)

TrTUP1 polyclonal antibody was raised against amino acids 203–216 (SPGPGRRGIGRPPN) (GenScript, Nanjing, China). For Co-IP analysis, *T*. *reesei* QM9414 strain were cultured in MA medium containing 1% Avicel and the collected mycelia were suspended in 50 mM HEPES lysis buffer at pH 7.5 plus 137 mM NaCl, 10% (v/v) glycerol, 1 mM PMSF (phenylme-thanesulfonyl fluoride), 1 μg/ml leupeptin, and 1 μg/ml pepstatin, and were broken with quartz sand on the Precellys 24 tissue homogenizer (Bertin Pharma, Montigny le Bretonneux, France). Co-IP assay was performed by incubating the anti-TrTUP1 antibody that was pre-coupled with protein A/G beads pre-coated with 1 mg/ml of bovine serum albumin (BSA), with cell free extract at 4°C for 2 h. Following IP and twice washes (10 min each time) with lysis buffer plus 500 mM NaCl, protein A/G beads were boiled with SDS-PAGE loading buffer followed by SDS-PAGE with a 5% stacking gel and a 10% separating gel running at 160 V for 1.5 h. IP trial without TrTUP1 antibody was used as a negative control. After silver staining, two extra visible bands corresponding to proteins co-immoniprecited with TrTUP1 antibody were excised from SDS gel and subjected to in-gel digestion with trypsin followed by mass spectrum analysis for identification using micrOTOF-QII mass spectrometer (Bruker, Karlsruhe, Germany).

### Recombinant *T*. *reesei* strains construction

To replace the native promoter of *Trtup1* with the *tcu1* gene promoter [34], the upstream 1761 bp fragment and downstream 1857 bp fragment flanking the start codon ATG of *Trtup1* gene were amplified from *T*. *reesei* genomic DNA, respectively, and ligated into the *Hin*dIII/*Asc*I and *Not*I/*Spe*I sites of pMDP_*tcu1*_-*pry4* [41] sequentially to obtain pMDP_*tcu1*_-*Trtup1*, which was subsequently linearized with *Spe*I and transformed into QM9414-Δ*pyr4* to obtain the P_*tcu1*_-*Trtup1* strain. *Trtup1* expression in P_*tcu1*_-*Trtup1* was significantly repressed when 20 mM coppper was present in the culture medium. To knock down *Trcyc8* expression using an RNA interference approach, a 774 bp fragment within the *Trcyc8* coding sequence and its reverse complemented sequence were amplified with *T*. *reesei* genomic DNA as template and respectively ligated into *Eco*RV/*Kpn*I and *Spe*I/*Not*I sites of the pKD-*hph* plasmid [40], to obtain pKD-*hph*-*Trcyc8*. After linearization with *Ssp*I, pKD-*hph*-*Trcyc8* was transformed into QM9414 to result in the P_*tcu1*_-*Trcyc8*^KD^ strain wherein *Trcyc8* expression was repressed without copper, but was rescued with exogenously adding 20 mM copper. To overexpress *xyr1*, the pMDP_*cdna1*_-*xyr1*-T_*trpC*_ plasmid [51]was transformed into QM9414-Δ*pyr4* to result in the OEX strain. In order to conditionally knock down *Trtup1* or *Trcyc8* in OEX, the linearized pMDP_*tcu1*_-*Trtup1* plasmid was transformed into OEX cells to obtain the OEX_P_*tcu1*_-*Trtup1* strain, while the linearized pKD-*hph*-*Trcyc8* plasmid was co-transformed with pFG1 [54] into OEX cells to obtain the OEX_P_*tcu1*_-*Trcyc8*^KD^ strain. *T*. *reesei* transformation was carried out essentially as previously described [55]. The transformants were selected on minimal media (MM) agar plates with 2% (w/v) glucose as carbon source for either uridine prototroph or for resistance to hygromycin (120 μg ml^-1^). All the *T*. *reesei* strains used in this study were listed in supplemental S1 Table.

### Vegetative growth and conidiation analyses

To analyze *T*. *reesei* vegetative growth, equal amounts of mycelia were inoculated on MM agar plates containing different carbon sources (glucose, glycerol, lactose, xylose, arabinose or Avicel) with or without exogenously adding copper and incubated at 30°C. To analyze *T*. *reesei* biomass on liquid MA medium containing 1% glucose, equal amounts of pre-cultured mycelia were inoculated and the mycelia collected at growth intervals were dried and weighed. For conidiation analysis, mycelia were inoculated on malt extract agar plates with or without 20 μM copper and incubated at 30°C for 5 days. The number of conidia was counted with a hemocytometer on an inverted optical microscope (Olympus, Tokyo, Japan).

### Quantitative RT-PCR

Total RNA was extracted using TRIzol reagent (Invitrogen, Grand Island, NY, USA) and purified using the TURBO DNA-free kit (Ambion, Austin, TX, USA) to remove genomic DNA according to the manufacturer’s instructions. Reverse transcription was carried out using the PrimeScript RT reagent Kit (Takara, Tokyo, Japan) according to the manufacturer’s instructions. Quantitative PCR was performed on LightCycler 480II (Roche, Basel, Switzerland) using the SYBR Green Supermix (Takara, Tokyo, Japan) according to the manufacturer’s instructions. Data analysis was performed using the comparative CT method. The endogenous *actin* gene was used as the control for normalization. All primers used for amplification in qRT-PCR assays were listed in S2 Table.

### Enzymatic activity analyses

Cellobiohydrolase and β-glucosidase activities were determined by measuring the amount of released *p*-nitrophenol using *p*-nitrophenyl-D-cellobioside (*p*NPC; Sigma, St. Louis, MO, USA) and *p*-nitrophenyl-β-D-glucopyranoside (*p*NPG; Sigma, St. Louis, MO, USA) as the substrates respectively. The assays were performed in 200 μl of reaction mixtures containing 50 μl of culture supernatant and 50 μl of the respective substrate plus 100 μl of 50 mM sodium acetate buffer (pH 4.8), and were then incubated at 45°C for 30 min. The reaction was stopped by addition of 50 μl of 10% Na_2_CO_3_ solution. One unit (U) of *p*NPCase or *p*NPGase activity is defined as the amount of enzyme releasing 1 μmol of *p*NP per minute [49]. SDS-PAGE and western blotting were performed according to standard protocols and CEL7A (CBH1) was immunoblotted using a polyclonal antibody raised against amino acids 426–446 of the protein, as previously described [55].

### Chromatin immunoprecipitation (ChIP)

ChIP assays were performed as previously described [49, 56]. Briefly, the mycelia were incubated in MA medium containing 1% formaldehyde at 30°C for 10 min with shaking before the cross-linking was quenched via adding 25 ml of 1.25 M glycine. The mycelia were then collected, suspended in 50 mM HEPES lysis buffer at pH 7.5 plus 150 mM NaCl, 1 mM EDTA, 0.5% Triton X-100, 0.1% sodium deoxycholate, 0.1% SDS, 1 mM PMSF, 1 μg/ml leupeptin, and 1 μg/ml pepstatin, and broken with glass beads (0.45 mm). Chromatin DNA was further sonicated to obtain sheared DNA fragments with an average size of approximately 500 bp. Immunoprecipitation with the antibody against XYR1, TrTUP1 and histone H4 was performed as previously described [56]. Quantitative PCR was performed on the precipitated chromatin DNAs using the same procedure with qRT-PCR. Relative enrichment of the DNAs was calculated as a percentage of the input DNA. All primers used for amplification in ChIP assays were listed in S2 Table. An excel file containing the original numerical data for ChIP-qPCR was included as S3 Data.

### Sequence analysis

Amino acid sequences from *T*. *reesei* and other relevant species were obtained from the NCBI (https://www.ncbi.nlm.nih.gov/) or JGI (https://genome.jgi.doe.gov/) database. The phylogenetic analyses of TrTUP1 and TrCYC8 were generated using the Neighbor-joining method with protein sequences aligned by ClustalW method based on the JTT with MEGA6 [57, 58]. Positions containing alignment gaps and missing data were pairwise deleted. Statistical confidence of the inferred phylogenetic relationships was assessed by performing 1000 bootstrap replicates. Protein sequence alignment was performed with the Multiple Sequence Alignment tool ClustalX and the protein domain was predicted via InterPro (http://www.ebi.ac.uk/interpro/) with the primary amino acid sequence.

### Statistical analysis

Statistical analysis was performed using the Student’s *t* test analysis. At least two or three biological replicates were performed for each analysis and the results and errors are the mean and SD, respectively, of these replicates.

## Supporting information

S1 Table *T*. *reesei* strains used in this research. (DOCX)

S2 Table Primers used in this research. (DOCX)

S1 Fig Protein sequence alignment of TrCYC8 and its homologs in *S*. *cerevisiae* and other filamentous fungi. Protein sequence alignment was performed by the Multiple Sequence Alignment tool ClustalX with the primary amino acid sequence of TrCYC8 and its homologs. The TPR domain was predicted via InterPro (http://www.ebi.ac.uk/interpro/) and labeled by red box as indicated.
(TIF)

S2 Fig Protein sequence alignment of TrTUP1 and its homologs in *S*. *cerevisiae* and other filamentous fungi. Protein sequence alignment was performed by the Multiple Sequence Alignment tool ClustalX with the primary amino acid sequence of TrTUP1 and its homologs. The WD40 and TUP1_N domains were predicted via InterPro (http://www.ebi.ac.uk/interpro/) and labeled by blue and red boxes as indicated, respectively.
(TIF)

S3 Fig Anchored PCR analyses of expected DNA integration in the P_*tcu1*_-*Trcyc8*^KD^ or P_*tcu1*_-*Trtup1* strain. (A) Schematic illustration of the *Trcyc8* knockdown expression cassette pKD-*hph*-*Trcyc8* and PCR analyses of its integration into the genome of P_*tcu1*_-*Trcyc8*^KD^ using primers as indicated. Lane 1–5, PCR products with genomic DNA of independent P_*tcu1*_-*Trcyc8*^KD^ transformants as template; PC, PCR product with pKD-*hph*-*Trcyc8* plasmid as template; Lane M, DNA molecular standard ladder. (B) Anchored PCR amplification to verify the replacement of the endogenous *Trtup1* promoter by the P_*tcu1*_ using the indicated primers. Lane 1–4, PCR products with genomic DNA of independent P_*tcu1*_-*Trtup1* transformants as template; NC and PC, PCR product obtained using the indicated primers with QM9414 genomic DNA as template; Lane M, DNA molecular standard ladder.
(TIF)

S4 Fig Knockdown of *Trcyc8* and *Trtup1* compromised xylanase expression induced by xylan. The culture supernatant of the QM9414, P_*tcu1*_-*Trcyc8*^KD^ and P_*tcu1*_-*Trtup1* strains cultured on 0.5% (w/v) xylan for the indicated time periods were assayed for xylanase activities. Significant differences (*t*-test *P<0.05, **P<0.01, ***P<0.001) were observed for the extracellular xylanase activities between QM9414 and P_*tcu1*_-*Trcyc8*^KD^ or P_*tcu1*_-*Trtup1* wherein *Trtup1* or *Trcyc8* was repressed.
(TIF)

S5 Fig PCR verification of integration of the *xyr1* expression cassette in the OEX, OEX_P_*tcu1*_-*Trcyc8*^KD^ and OEX_P_*tcu1*_-*Trtup1* strains. (A) PCR amplification to verify integration of the pMDP_*cdna1*_-*xyr1*-T_*trpC*_ plasmid in the OEX strain. Lane 1–3, PCR products with genomic DNA of independent OEX transformants as template; NC, PCR product with QM9414 genomic DNA as template; Lane M, DNA molecular standard ladder. (B) PCR analyses of integration of the *Trcyc8* knockdown expression cassette into the OEX_P_*tcu1*_-*Trcyc8*^KD^ genome using the two primer pairs as indicated in S3A Fig. Lane 1–3, PCR products with genomic DNA of independent OEX_P_*tcu1*_-*Trcyc8*^KD^ transformants as template; PC, PCR product with the pKD-*hph*-*Trcyc8* plasmid as template; Lane M, DNA molecular standard ladder. (C) PCR amplification to verify the replacement of the endogenous *Trtup1* promoter by the P_*tcu1*_ in OEX_P_*tcu1*_-*Trtup1* using the primer pairs as indicated in S3B Fig. Lane 1–3, PCR products with genomic DNA of independent OEX_P_*tcu1*_-*Trtup1* transformants as template; NC and PC, PCR product obtained using the indicated primers with QM9414 genomic DNA as template, respectively; Lane M, DNA molecular standard ladder.
(TIF)

S1 Data Peptide information of TrTUP1 from mass spectrum analysis. (XLSX)

S2 Data Peptide information of TrCYC8 from mass spectrum analysis. (XLSX)

S3 Data ChIP-qPCR original numerical data for Figs 6, 8 and 9. (XLSX)
