# Supplementary material for: Interdependent recruitment of CYC8/TUP1 and the transcriptional activator XYR1 at target promoters is required for induced cellulase gene expression in Trichoderma reesei
Source: PLoS Genet. 2021 Feb 19;17(2):e1009351. doi: 10.1371/journal.pgen.1009351 (PMC7894907; doi:10.1371/journal.pgen.1009351)
Supplement: S1 Table — (DOCX) [file pgen.1009351.s001.docx]

**S1 Table. *T. reesei* strains used in this research**

| **Strain name** | **Genotype and** **Characteristics** | **Reference** |
| --- | --- | --- |
| QM9414 | cellulase higher producer derivative from the wild type strain QM6a | ATCC 26921 |
| QM9414-Δ*pyr4* | Deleting the uridine trophic marker gene *pyr4* in QM9414 | [1] |
| Δ*xyr1* | Deleting the *xyr1* in QM9414 | [2] |
| OEX  P*_tcu1_*-*Trtup1*  P*_tcu1_*-*Trcyc8*^KD^ | Constitutive expression of XYR1 in QM9414-Δ*pyr4*  Replacing endogenous *Trtup1* promoter using P*_tcu1_* promoter in QM9414-Δ*pyr4*; *Trtup1* expression is repressed when the 20 mM copper is present in the media but it is overexpressed when the copper is absent.  Knockdown of *Trcyc8* in QM9414; *Trcyc8* expression is repressed in the media without copper but its expression is rescued when the 20 mM copper is present. | This study  This study  This study |
| OEX_ P*_tcu1_*-*Trtup1* | Replacing endogenous *Trtup1* promoter using P*_tcu1_* promoter in OEX; *Trtup1* expression is repressed when the 20 mM copper is present in the media but it is overexpressed when the copper is absent. | This study |
| OEX_ P*_tcu1_*-*Trcyc8*^KD^ | Knockdown of *Trcyc8* in OEX; *Trcyc8* expression is repressed in the media without copper but its expression is rescued when the 20 mM copper is present. | This study |

**References**

1. Wang L, Zheng F, Zhang W, Zhong Y, Chen G, et al. (2018) A copper-controlled RNA interference system for reversible silencing of target genes in Trichoderma reesei. Biotechnol Biofuels 11: 33.

2. Stricker AR, Grosstessner-Hain K, Wurleitner E, Mach RL (2006) Xyr1 (xylanase regulator 1) regulates both the hydrolytic enzyme system and D-xylose metabolism in Hypocrea jecorina. Eukaryot Cell 5: 2128-2137.
