# Supplementary material for: Interdependent recruitment of CYC8/TUP1 and the transcriptional activator XYR1 at target promoters is required for induced cellulase gene expression in Trichoderma reesei
Source: PLoS Genet. 2021 Feb 19;17(2):e1009351. doi: 10.1371/journal.pgen.1009351 (PMC7894907; doi:10.1371/journal.pgen.1009351)
Supplement: S2 Table — (DOCX) [file pgen.1009351.s002.docx]

**S2 Table. Primers used in this research**

RT-qPCR

| *cel7a*_qF | CTTGGCAACGAGTTCTCTT |
| --- | --- |
| *cel7a*_qR | TGTTGGTGGGATACTTGCT |
| *cel7b*_qF | CGGCTACAAAAGCTACTACG |
| *cel7b*_qR | CTGGTACTTGCGGGTGAT |
| *cel3a*_qF | CGAAGCACTATATCCTCAAC |
| *cel3a*_qR | GTATTGACCTTGTTGTACGA |
| *xyr1*_qF | CCATCAACCTTCTAGACGAC |
| *xyr1*_qR | AACCCTGCAGGAGATAGAC |
| *cyc8*_qF | GAGGAACCCGTGTCCAAGAA |
| *cyc8*_qR | CAGCCTCGGAAGGATGGTAG |
| *tup1*_qF | CGAACTATCCGCAACCACTT |
| *tup1*_qR | GACACGAACGCTCTTATCCAA |
| *actin*_qF | TGAGAGCGGTGGTATCCACG |
| *actin*_qR | GGTACCACCAGACATGACAATGTTG |

ChIP-qPCR

| P*cel7a*_qF | GGCAGTGATGGAAGACAGTGAAA |
| --- | --- |
| P*cel7a*_qR | TCGTCGTATCGGCAGACAAACCT |
| P*cel7b*_qF | CTGGACCAATCAGGCTAAATC |
| P*cel7b*_qR | TGTTTCGAGCCGTTATACAGAC |
| P*cel3a*_qF | GGCAGGTCGATTCTCGGTAAGT |
| P*cel3a*_qR | GACAAGAAGCCAGCCGAGGG |
| P*actin*_qF | CATCGTGGCAGCGGAGTTA |
| P*actin*_qR | TTGAAGAGGGCGAAGATAGACA |
