# Supplementary figures and images for: Interdependent recruitment of CYC8/TUP1 and the transcriptional activator XYR1 at target promoters is required for induced cellulase gene expression in Trichoderma reesei
Source: PLoS Genet. 2021 Feb 19;17(2):e1009351. doi: 10.1371/journal.pgen.1009351 (PMC7894907; doi:10.1371/journal.pgen.1009351)

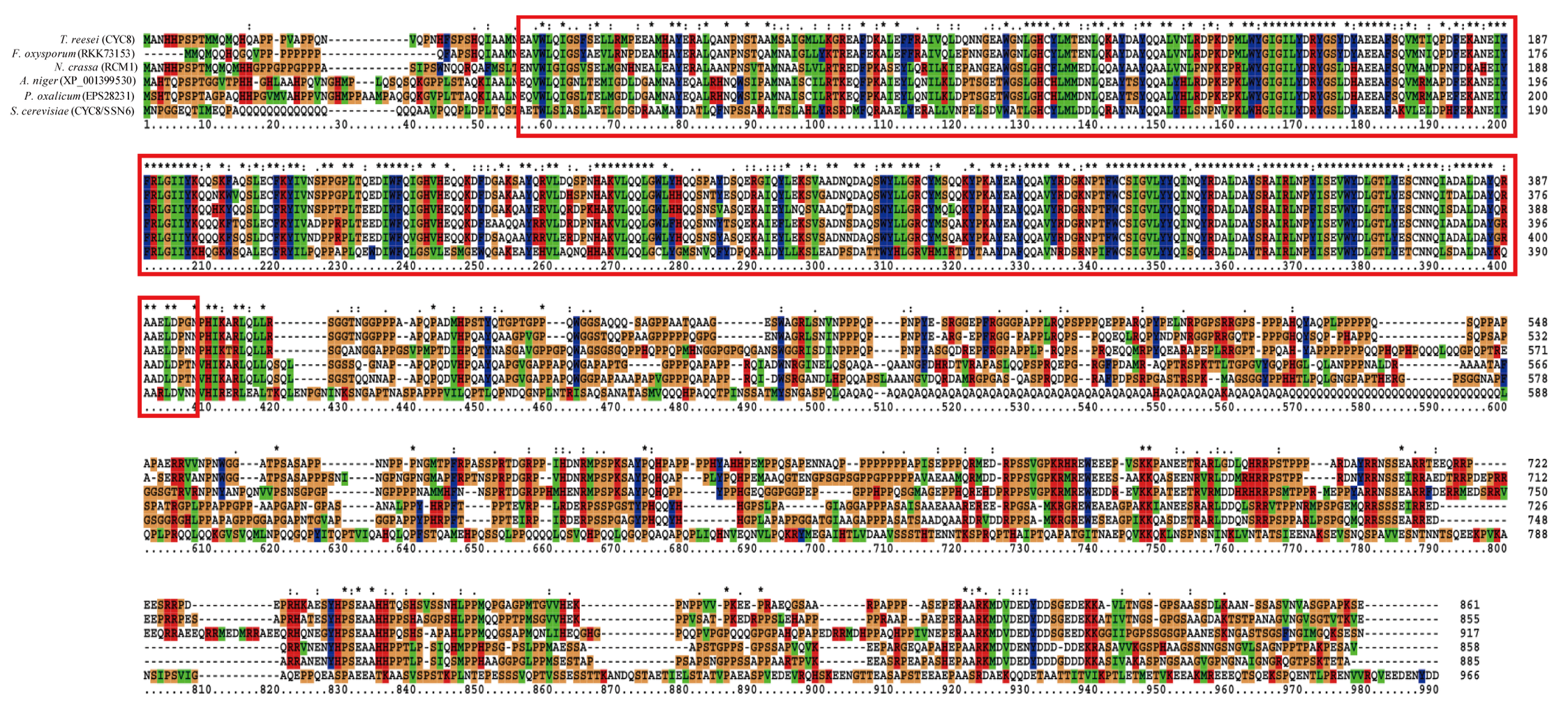

Supplement: S1 Fig — Protein sequence alignment was performed by the Multiple Sequence Alignment tool ClustalX with the primary amino acid sequence of TrCYC8 and its homologs. The TPR domain was predicted via InterPro (http://www.ebi.ac.uk/interpro/) and labeled by red box as indicated. (TIF) [file pgen.1009351.s003.tif]

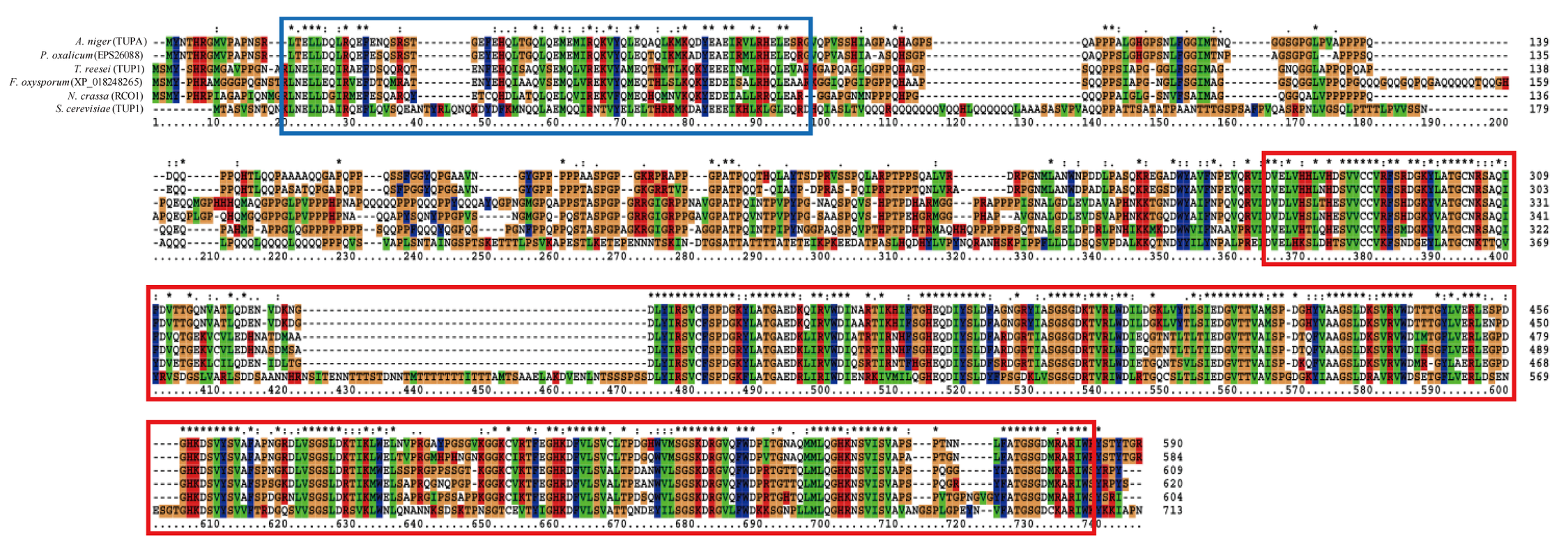

Supplement: S2 Fig — Protein sequence alignment was performed by the Multiple Sequence Alignment tool ClustalX with the primary amino acid sequence of TrTUP1 and its homologs. The WD40 and TUP1_N domains were predicted via InterPro (http://www.ebi.ac.uk/interpro/) and labeled by blue and red boxes as indicated, respectively. (TIF) [file pgen.1009351.s004.tif]

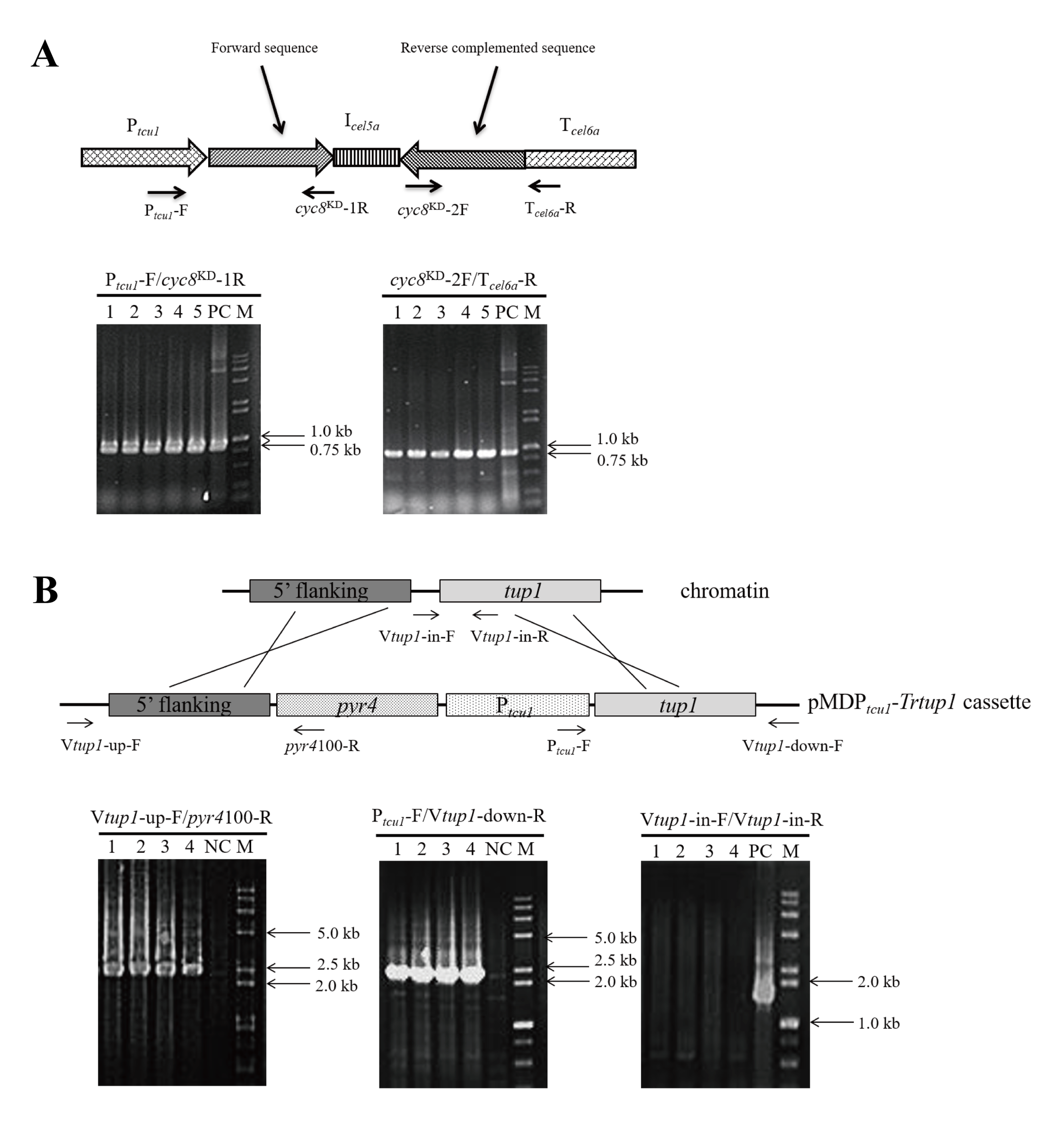

Supplement: S3 Fig — (A) Schematic illustration of the Trcyc8 knockdown expression cassette pKD-hph-Trcyc8 and PCR analyses of its integration into the genome of Ptcu1-Trcyc8KD using primers as indicated. Lane 1–5, PCR products with genomic DNA of independent Ptcu1-Trcyc8KD transformants as template; PC, PCR product with pKD-hph-Trcyc8 plasmid as template; Lane M, DNA molecular standard ladder. (B) Anchored PCR amplification to verify the replacement of the endogenous Trtup1 promoter by the Ptcu1 using the indicated primers. Lane 1–4, PCR products with genomic DNA of independent Ptcu1-Trtup1 transformants as template; NC and PC, PCR product obtained using the indicated primers with QM9414 genomic DNA as template; Lane M, DNA molecular standard ladder. (TIF) [file pgen.1009351.s005.tif]

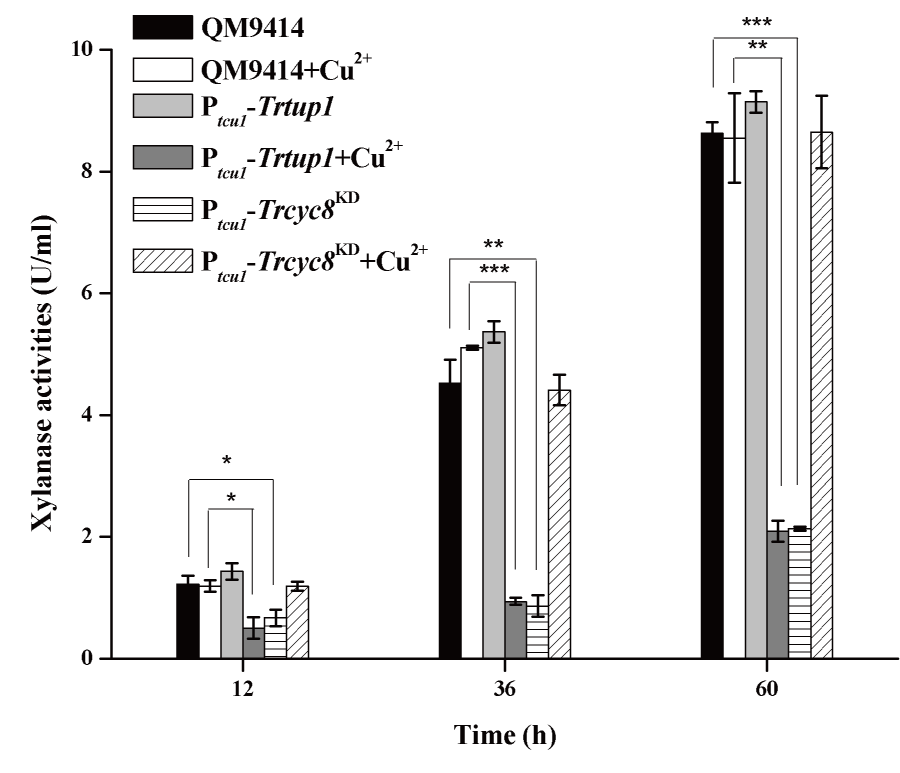

Supplement: S4 Fig — The culture supernatant of the QM9414, Ptcu1-Trcyc8KD and Ptcu1-Trtup1 strains cultured on 0.5% (w/v) xylan for the indicated time periods were assayed for xylanase activities. Significant differences (t-test *P<0.05, **P<0.01, ***P<0.001) were observed for the extracellular xylanase activities between QM9414 and Ptcu1-Trcyc8KD or Ptcu1-Trtup1 wherein Trtup1 or Trcyc8 was repressed. (TIF) [file pgen.1009351.s006.tif]

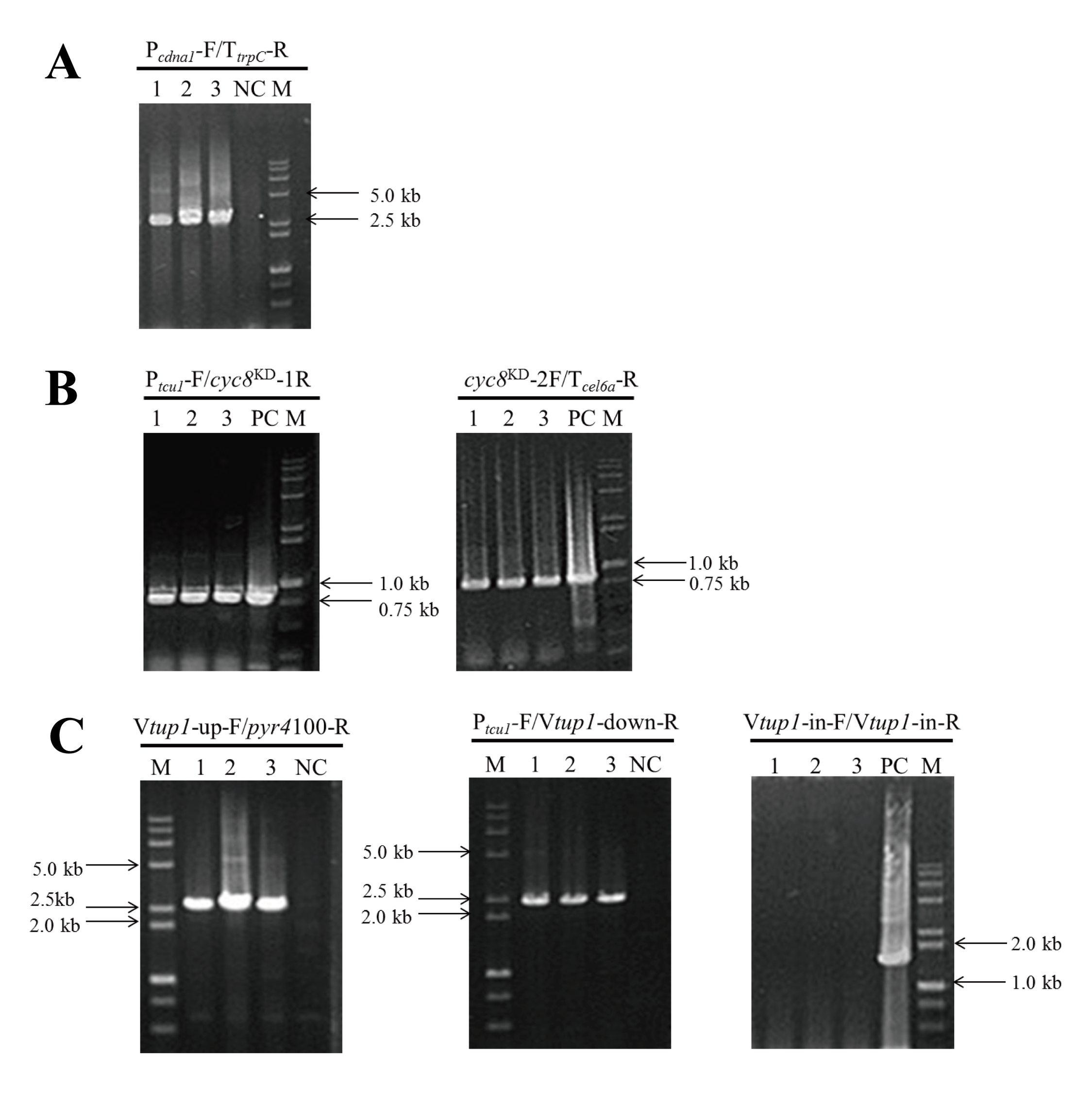

Supplement: S5 Fig — (A) PCR amplification to verify integration of the pMDPcdna1-xyr1-TtrpC plasmid in the OEX strain. Lane 1–3, PCR products with genomic DNA of independent OEX transformants as template; NC, PCR product with QM9414 genomic DNA as template; Lane M, DNA molecular standard ladder. (B) PCR analyses of integration of the Trcyc8 knockdown expression cassette into the OEX_Ptcu1-Trcyc8KD genome using the two primer pairs as indicated in S3A Fig. Lane 1–3, PCR products with genomic DNA of independent OEX_Ptcu1-Trcyc8KD transformants as template; PC, PCR product with the pKD-hph-Trcyc8 plasmid as template; Lane M, DNA molecular standard ladder. (C) PCR amplification to verify the replacement of the endogenous Trtup1 promoter by the Ptcu1 in OEX_Ptcu1-Trtup1 using the primer pairs as indicated in S3B Fig. Lane 1–3, PCR products with genomic DNA of independent OEX_Ptcu1-Trtup1 transformants as template; NC and PC, PCR product obtained using the indicated primers with QM9414 genomic DNA as template, respectively; Lane M, DNA molecular standard ladder. (TIF) [file pgen.1009351.s007.tif]
